# Supplementary material for: Loss of PHF6 causes spontaneous seizures, enlarged brain ventricles and altered transcription in the cortex of a mouse model of the Börjeson–Forssman–Lehmann intellectual disability syndrome
Source: PLoS Genet. 2024 Oct 15;20(10):e1011428. doi: 10.1371/journal.pgen.1011428 (PMC11478892; doi:10.1371/journal.pgen.1011428)
Supplement: S1 Table — (PDF) [file pgen.1011428.s001.pdf]

S1 Table: *PHF6* mutations and references for the BFLS samples shown in Fig 1

Lane	DNA mutation (c.)	Protein mutation (p.)	(family number)+ Reference
5	134G>A	C45Y	(4) [1]
6	296G>T	C99F	(2) [1]
7	999_1001delTGA	D333del	[2]
8	1009_1011delGAA	E337del	[3]
9	1009_1011delGAA	E337del	[3]
10	1024C>T	R342*	(1) [1]
11	1024C>T	R342*	(3) [4]
12	1024C>T	R342*	(1) [1]
13	27dup	G10fs*12	[5]

+Family numbers in brackets refer to the number given in the original reference.

References

1. Lower KM, Turner G, Kerr BA, Mathews KD, Shaw MA, Gedeon AK, et al. Mutations in PHF6 are associated with Borjeson-Forssman-Lehmann syndrome. Nat Genet. 2002;32(4):661-5. doi: 10.1038/Ng1040. PubMed PMID: WOS:000179593000021.
2. Baumstark A, Lower KM, Sinkus A, Andriuskeviciute I, Jurkeniene L, Gecz J, et al. Novel PHF6 mutation p.D333del causes Borjeson-Forssman-Lehmann syndrome. Journal of Medical Genetics. 2003;40(4):e50. doi: Artn E50 Doi 10.1136/Jmg.40.4.E50. PubMed PMID: WOS:000182155900032.
3. Mücke J, Just W. Das Börjeson-Forssman-Lehmann Syndrom (BFLS). Monatsschr Kinderheilkd. 2007;155((Suppl1)). doi: https://doi.org/10.1007/s00112-005-1154-x.
4. Lower KM, Solders G, Bondeson ML, Nelson J, Brun A, Crawford J, et al. 1024C > T (R342X) is a recurrent PHF6 mutation also found in the original Borjeson-Forssman-Lehmann syndrome family. European Journal of Human Genetics. 2004;12(10):787-9. doi: Doi 10.1038/Sj.Ejhg.5201228. PubMed PMID: WOS:000223928700001.
5. Crawford J, Lower KM, Hennekam RCM, Van Esch H, Megarbane A, Lynch SA, et al. Mutation screening in Borjeson-Forssman-Lehmann syndrome: identification of a novel de novo PHF6 mutation in a female patient. J Med Genet. 2006;43(3):238-43. doi: 10.1136/Jmg.2005.033084. PubMed PMID: WOS:000235890800008.
