# Supplementary material for: Loss of PHF6 causes spontaneous seizures, enlarged brain ventricles and altered transcription in the cortex of a mouse model of the Börjeson–Forssman–Lehmann intellectual disability syndrome
Source: PLoS Genet. 2024 Oct 15;20(10):e1011428. doi: 10.1371/journal.pgen.1011428 (PMC11478892; doi:10.1371/journal.pgen.1011428)
Supplement: S2 Table — (PDF) [file pgen.1011428.s002.pdf]

S2 Table: Effect of *Phf6* germline mutation on survival on the C57BL/6 background

Age	Females				Males				Total number	p-value for *Phf6*$^{-/Y}$ males	p-value for *Phf6*$^{+/-}$ females
	Phf6$^{+/+}$		*Phf6*$^{+/-}$		*Phf6*$^{+/Y}$		*Phf6*$^{-/Y}$				
Post-natal day 0	57	27%	57	27%	96	46%	0	0%	210	$< 10^{-6}$	0.79
Weaning (3 weeks)	54	31%	42	24%	79	45%	0	0%	175	$< 10^{-6}$	0.42

The number and percentage of each genotype from crosses between *Phf6*$^{+/-}$ heterozygous females with *Phf6*$^{+/Y}$ males is shown. The expected frequency of each genotype is 25%. Data were analysed by a binomial test. P-values are shown for the representation of the *Phf6*$^{-/Y}$ and *Phf6*$^{+/-}$ genotypes.
