# Supplementary material for: Loss of PHF6 causes spontaneous seizures, enlarged brain ventricles and altered transcription in the cortex of a mouse model of the Börjeson–Forssman–Lehmann intellectual disability syndrome
Source: PLoS Genet. 2024 Oct 15;20(10):e1011428. doi: 10.1371/journal.pgen.1011428 (PMC11478892; doi:10.1371/journal.pgen.1011428)
Supplement: S1 Fig — Time to first seizure in a small number of females with homozygous deletion of Phf6 in the nervous system. The median latency was 400 days. Controls are the same cohort shown in Fig 2B. n = 11 Phf6+/+;Nes-creTg/+ mice, n = 4 Phf6lox/lox;Nes-creTg/+ mice. Data were analysed by a log-rank (Mantel-Cox) test. (PDF) [file pgen.1011428.s006.pdf]

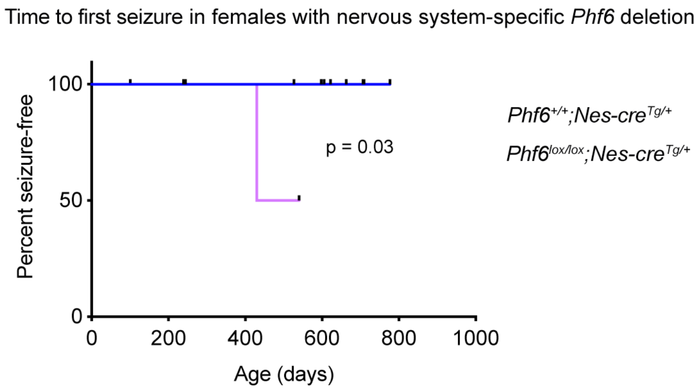

S1 Fig: Females with homozygous *Phf6* deletion in the central nervous system are susceptible to seizures

Time to first seizure in a small number of females with homozygous deletion of *Phf6* in the nervous system. The median latency was 400 days. Controls are the same cohort shown in Fig 2B. n = 11 *Phf6*$^{+/+}$*;Nes-cre*$^{Tg/+}$ mice, n = 4 *Phf6*$^{lox/lox}$*;Nes-cre*$^{Tg/+}$ mice.
Data were analysed by a log-rank (Mantel-Cox) test.
