# Supplementary material for: Loss of PHF6 causes spontaneous seizures, enlarged brain ventricles and altered transcription in the cortex of a mouse model of the Börjeson–Forssman–Lehmann intellectual disability syndrome
Source: PLoS Genet. 2024 Oct 15;20(10):e1011428. doi: 10.1371/journal.pgen.1011428 (PMC11478892; doi:10.1371/journal.pgen.1011428)
Supplement: S2 Fig — (A) Representative sections of N = 3 Phf6+/Y;Nes-creTg/+ and 3 Phf6lox/Y;Nes-creTg/+ adult brains (13–14 weeks old) stained with anti-GAD67 antibody by immunohistochemistry. Scale bar equals 100 μm. The schematic on the right indicates the approximate locations where GAD67+ cells were quantified in sections in the left and right hemisphere on each cortex section. (B) Enumeration of GAD67+ cells per mm2 of cortex section. The average ± sem for each genotype is shown with circles representing data from individual animals. The number of GAD67+ cells was counted on matched sections of the parietal cortex and divided by the area analysed. Data were analysed by two-tailed Student’s t-test (p = 0.51). (PDF) [file pgen.1011428.s007.pdf]

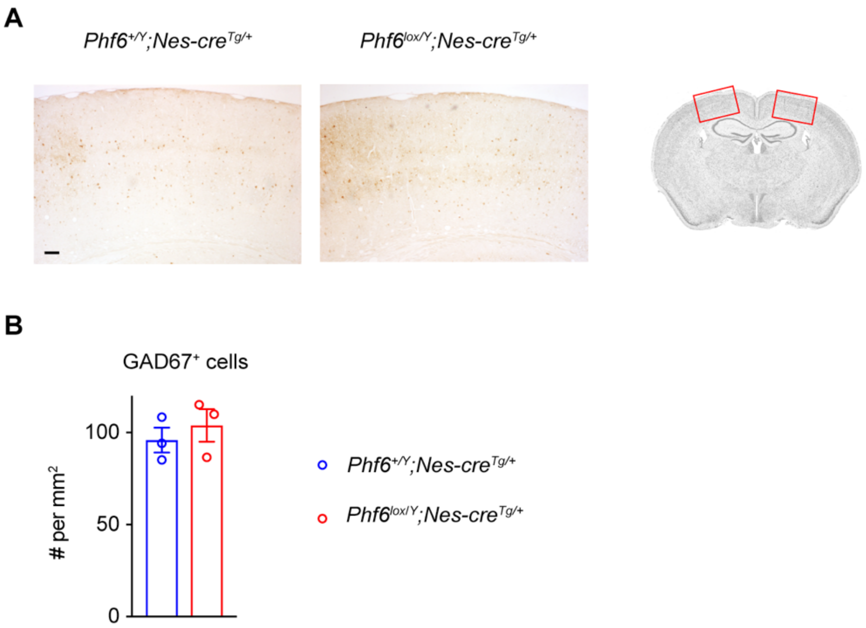

S2 Fig: No change in GAD67^{+} neurons in *Phf6*-deleted mice

(A) Representative sections of N = 3 *Phf6$^{+/Y}$;Nes-cre$^{Tg/+}$* and 3 *Phf6$^{lox/Y}$;Nes-cre$^{Tg/+}$* adult brains (13-14 weeks old) stained with anti-GAD67 antibody by immunohistochemistry. Scale bar equals 100 µm. The schematic on the right indicates the approximate locations where GAD67^{+} cells were quantified in sections in the left and right hemisphere on each cortex section.

(B) Enumeration of GAD67^{+} cells per mm^2 of cortex section. The average ± sem for each genotype is shown with circles representing data from individual animals. The number of GAD67^{+} cells was counted on matched sections of the parietal cortex and divided by the area analysed. Data were analysed by two-tailed Student's t-test ($p = 0.51$).
