# Supplementary material for: Loss of PHF6 causes spontaneous seizures, enlarged brain ventricles and altered transcription in the cortex of a mouse model of the Börjeson–Forssman–Lehmann intellectual disability syndrome
Source: PLoS Genet. 2024 Oct 15;20(10):e1011428. doi: 10.1371/journal.pgen.1011428 (PMC11478892; doi:10.1371/journal.pgen.1011428)
Supplement: S3 Fig — Representative images showing matched 10 μm brain sections of a Phf6lox/Y;Nes-creTg/+ and a Phf6+/Y;Nes-creTg/+ brain stained with cresyl violet. Serial sections of N = 3 Phf6lox/Y;Nes-creTg/+ mice 17–37 days after onset of seizures and 3 age-matched Phf6+/Y;Nes-creTg/+ control mice (394 to 483 days old) were examined. Scale bar = 1 mm. (PDF) [file pgen.1011428.s008.pdf]

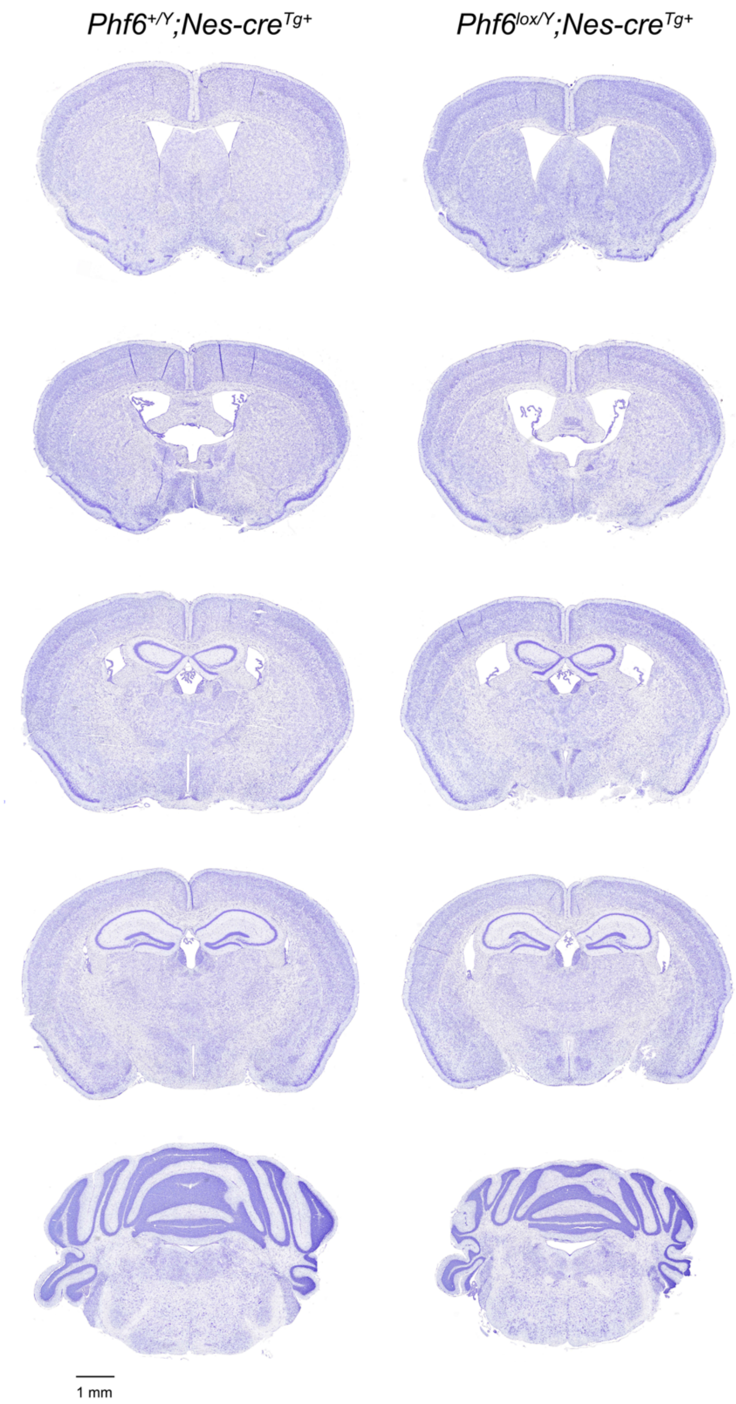

S3 Fig: Histology of adult seizure-affected *Phf6*$^{lox/Y}$*;Nes-cre*$^{Tg/+}$ and *Phf6*$^{+/Y;}$*Nes-cre*$^{Tg/+}$ control brains

Representative images showing matched 10 µm brain sections of a *Phf6*$^{lox/Y}$*;Nes-cre*$^{Tg/+}$ and a *Phf6*$^{+/Y}$*;Nes-cre*$^{Tg/+}$ brain stained with cresyl violet. Serial sections of N = 3 *Phf6*$^{lox/Y}$*;Nes-cre*$^{Tg/+}$ mice 17-37 days after onset of seizures and 3 age-matched *Phf6*$^{+/Y}$*;Nes-cre*$^{Tg/+}$ control mice (394 to 483 days old) were examined.

Scale bar = 1 mm.
