# Supplementary material for: Loss of PHF6 causes spontaneous seizures, enlarged brain ventricles and altered transcription in the cortex of a mouse model of the Börjeson–Forssman–Lehmann intellectual disability syndrome
Source: PLoS Genet. 2024 Oct 15;20(10):e1011428. doi: 10.1371/journal.pgen.1011428 (PMC11478892; doi:10.1371/journal.pgen.1011428)
Supplement: S4 Fig — (A) Representative image of the cerebellum of N = 3 Phf6+/Y;Nes-creTg/+ and 3 Phf6lox/Y;Nes-creTg/+ brain. Scale bar = 200 μm. (B) Quantification of the number of Purkinje cells in the cerebellum in three mice per genotype (aged 394 to 483 days old). Cells were counted in 4 evenly spaced sections per animal. Data are presented as the number of Purkinje cells per length of the Purkinje cell layer in mm showing the results from individual animals as a circle and the mean ± sem for each genotype. Data were analysed using a two-tailed Student’s t-test (p = 0.17). (PDF) [file pgen.1011428.s009.pdf]

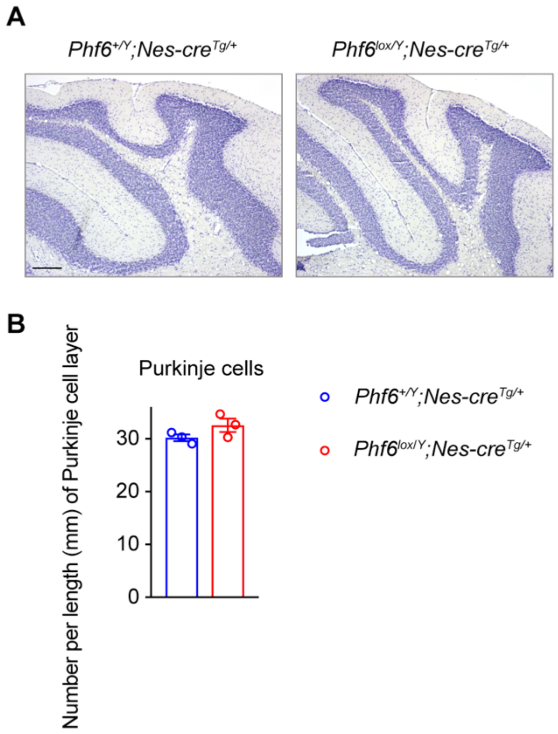

S4 Fig: The number of Purkinje cells of the cerebellum is not affected by loss of PHF6

(A) Representative image of the cerebellum of N = 3 *Phf6*$^{+/Y}$*;Nes-cre*$^{Tg/+}$ and 3 *Phf6*$^{lox/Y}$*;Nes-cre*$^{Tg/+}$ brain. Scale bar = 200 µm.

(B) Quantification of the number of Purkinje cells in the cerebellum in three mice per genotype (aged 394 to 483 days old). Cells were counted in 4 evenly spaced sections per animal. Data are presented as the number of Purkinje cells per length of the Purkinje cell layer in mm showing the results from individual animals as a circle and the mean ± sem for each genotype. Data were analysed using a two-tailed Student's t-test ($p = 0.17$).
