# Supplementary material for: Loss of PHF6 causes spontaneous seizures, enlarged brain ventricles and altered transcription in the cortex of a mouse model of the Börjeson–Forssman–Lehmann intellectual disability syndrome
Source: PLoS Genet. 2024 Oct 15;20(10):e1011428. doi: 10.1371/journal.pgen.1011428 (PMC11478892; doi:10.1371/journal.pgen.1011428)
Supplement: S5 Fig — (A) Volume of the cerebral cortex divided by the volume of the total brain in the sections surrounding the lateral ventricles is slightly reduced in Phf6lox/Y;Nes-creTg/+ vs. Phf6+/Y;Nes-creTg/+ brains. (B) Volume of the rest of the brain (i.e., minus the cortex and lateral ventricles), divided by the volume of the total brain in the sections surrounding the lateral ventricles in Phf6lox/Y;Nes-creTg/+ vs. Phf6+/Y;Nes-creTg/+ brains. LV = lateral ventricles. (C) Depth of individual cortical layers as percentage of total cortex depth in Phf6lox/Y;Nes-creTg/+ vs. Phf6+/Y;Nes-creTg/+ brains. N = 3 Phf6lox/Y;Nes-creTg/+ vs. 3 Phf6+/Y;Nes-creTg/+ mice (aged 394 to 483 days old). Circles represent individual animals (A-C). Lines between individual data points join each Phf6lox/Y;Nes-creTg/+ animal to its paired control (A,B). Data (C) are presented as mean ± sem. Data were analyzed by a paired t-test (A,B) or unpaired two-tailed t-test (C). (PDF) [file pgen.1011428.s010.pdf]

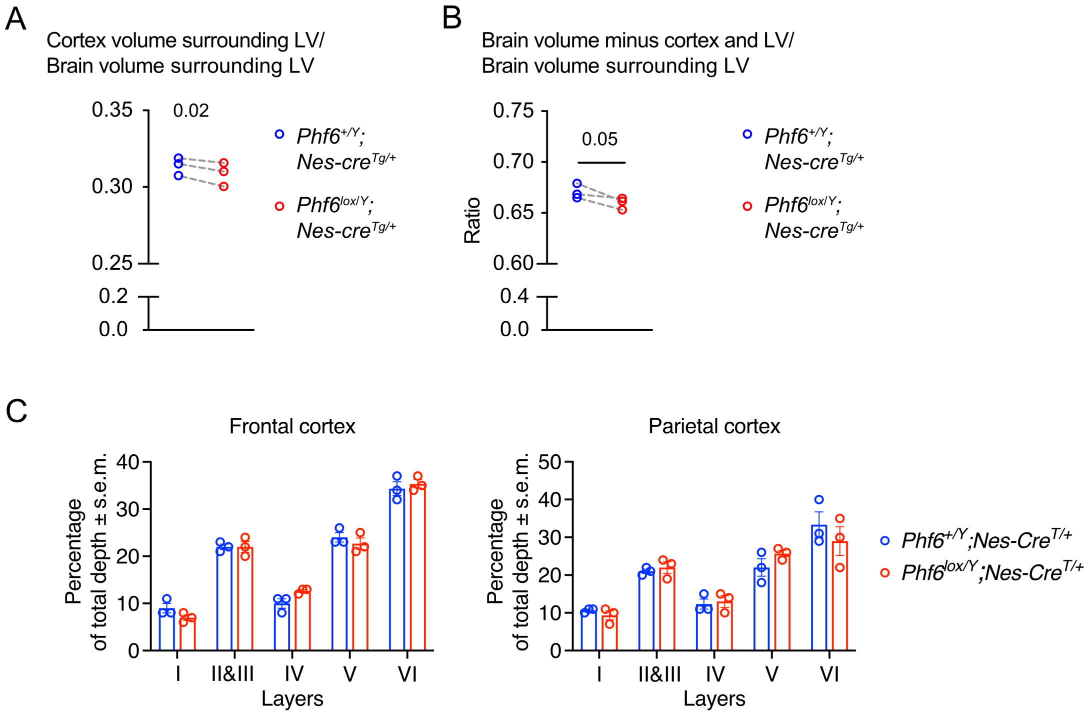

S5 Fig: Reduction in cortex volume and other regions of the brain caused by loss of PHF6

(A) Volume of the cerebral cortex divided by the volume of the total brain in the sections surrounding the lateral ventricles is slightly reduced in *Phf6*$^{lox/Y}$*;Nes-cre*$^{Tg/+}$vs. *Phf6*$^{+/Y}$*;Nes-cre*$^{Tg/+}$ brains.

(B) Volume of the rest of the brain (i.e., minus the cortex and lateral ventricles), divided by the volume of the total brain in the sections surrounding the lateral ventricles in *Phf6*$^{lox/Y}$*;Nes-cre*$^{Tg/+}$ vs. *Phf6*$^{+/Y}$*;Nes-cre*$^{Tg/+}$ brains.

LV = lateral ventricles.

(C) Depth of individual cortical layers as percentage of total cortex depth in *Phf6*$^{lox/Y}$*;Nes-cre*$^{Tg/+}$ vs. *Phf6*$^{+/Y}$*;Nes-cre*$^{Tg/+}$ brains.

N = 3 *Phf6*$^{lox/Y}$*;Nes-cre*$^{Tg/+}$vs. 3 *Phf6*$^{+/Y}$*;Nes-cre*$^{Tg/+}$ mice (aged 394 to 483 days old). Circles represent individual animals (A-C). Lines between individual data points join each *Phf6*$^{lox/Y}$*;Nes-cre*$^{Tg/+}$ animal to its paired control (A,B). Data (C) are presented as mean ± sem. Data were analyzed by a paired t-test (A,B) or unpaired two-tailed t-test (C).
