# Supplementary material for: Loss of PHF6 causes spontaneous seizures, enlarged brain ventricles and altered transcription in the cortex of a mouse model of the Börjeson–Forssman–Lehmann intellectual disability syndrome
Source: PLoS Genet. 2024 Oct 15;20(10):e1011428. doi: 10.1371/journal.pgen.1011428 (PMC11478892; doi:10.1371/journal.pgen.1011428)
Supplement: S6 Fig — (A) Representative images of cresyl violet stained paraffin section of Phf6lox/Y;Nes-creTg/+ vs. Phf6+/Y;Nes-creTg/+ 13–14-week-old mouse brains at the level of the parietal cortex. Scale bar = 1 mm. (B) Assessment of the total brain area and the relative area of the lateral ventricles at the level of the frontal cortex and the 3rd ventricle at the level of the parietal cortex. The ventricle area is expressed as percentage of the total brain tissue area in the same section. Four sections per animal, two per brain region at the level of the frontal and parietal cortex were assessed. The left and right lateral ventricle were assessed. N = N = 3 Phf6lox/Y;Nes-creTg/+ vs. 3 Phf6+/Y;Nes-creTg/+ 13–14-week-old mice. Data are displayed as mean ± sem and were analysed by two-way ANOVA (B, left) and unpaired, two-tailed Student’s t test (B, middle and right). (PDF) [file pgen.1011428.s011.pdf]

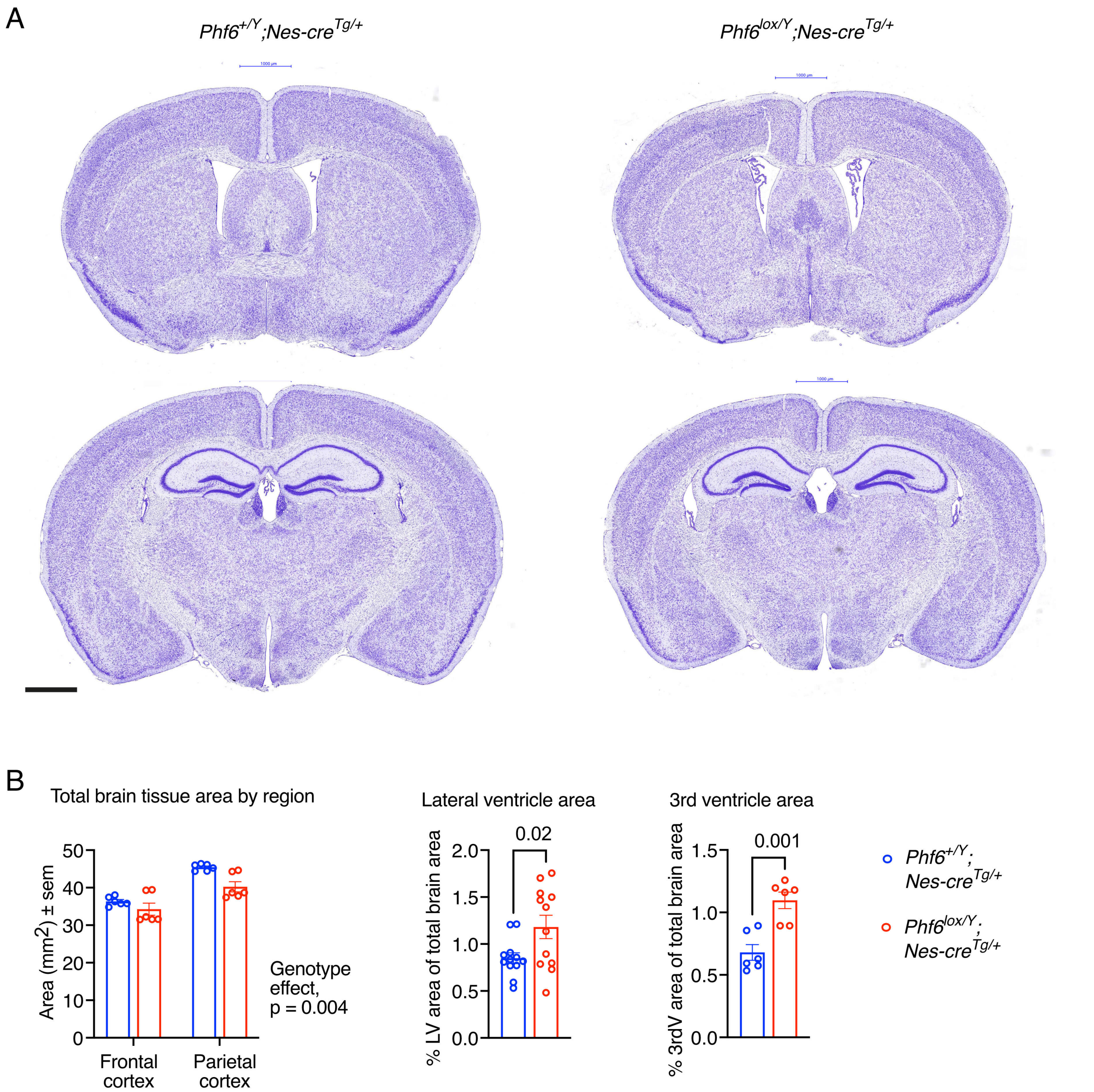

S6 Fig: Assessment of ventricle area in brain sections of 13–14-week-old mice prior to the onset of seizures

(A) Representative images of cresyl violet stained paraffin section of *Phf6*$^{lox/Y}$;*Nes-cre*$^{Tg/+}$ vs. *Phf6*$^{+/Y}$;*Nes-cre*$^{Tg/+}$ 13–14-week-old mouse brains at the level of the parietal cortex. Scale bar = 1 mm.

(B) Assessment of the total brain area and the relative area of the lateral ventricles at the level of the frontal cortex and the 3rd ventricle at the level of the parietal cortex. The ventricle area is expressed as percentage of the total brain tissue area in the same section. Four sections per animal, two per brain region at the level of the frontal and parietal cortex were assessed. The left and right lateral ventricle were assessed.

N = N = 3 *Phf6*$^{lox/Y}$;*Nes-cre*$^{Tg/+}$ vs. 3 *Phf6*$^{+/Y}$;*Nes-cre*$^{Tg/+}$ 13–14-week-old mice. Data are displayed as mean ± sem and were analysed by two-way ANOVA (B, left) and unpaired, two-tailed Student's t test (B, middle and right).
