# Supplementary material for: Loss of PHF6 causes spontaneous seizures, enlarged brain ventricles and altered transcription in the cortex of a mouse model of the Börjeson–Forssman–Lehmann intellectual disability syndrome
Source: PLoS Genet. 2024 Oct 15;20(10):e1011428. doi: 10.1371/journal.pgen.1011428 (PMC11478892; doi:10.1371/journal.pgen.1011428)
Supplement: S7 Fig — (A) Incidence of hydrocephalus with enlarged skull of N = 205 female Phf6+/+ and 268 female Phf6+/– mice. (B) Incidence of hydrocephalus with enlarged skull of N = 104 female Phf6+/+;Nes-creTg/+ and 101 female Phf6+/lox;Nes-creTg/+. No significant difference was detected. (C) Incidence of hydrocephalus with enlarged skull of N = 109 male Phf6+/Y;Nes-creTg/+ and 74 male Phf6lox/Y;Nes-creTg/+. No significant difference was detected. Checked points indicate mice that were euthanized for reasons other than hydrocephalus. Data were analysed by Mantel-Cox log-rank test. (PDF) [file pgen.1011428.s012.pdf]

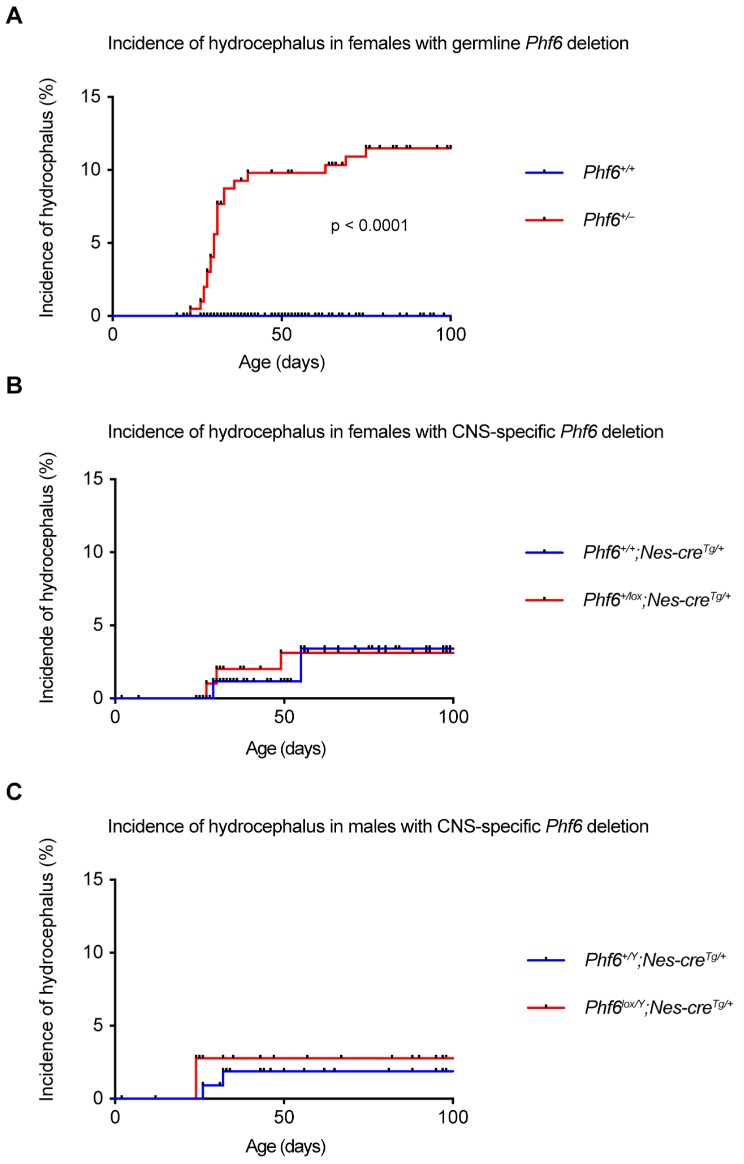

S7 Fig: Effect of *Phf6* mutation on the incidence hydrocephalus with enlarged skull

(A) Incidence of hydrocephalus with enlarged skull of N = 205 female *Phf6*$^{+/+}$ and 268 female *Phf6*$^{+/-}$ mice.

(B) Incidence of hydrocephalus with enlarged skull of N = 104 female *Phf6*$^{+/+}$*;Nes-cre*$^{Tg/+}$ and 101 female *Phf6*$^{+/lox}$*;Nes-cre*$^{Tg/+}$. No significant difference was detected.

(C) Incidence of hydrocephalus with enlarged skull of N = 109 male *Phf6*$^{+/Y}$*;Nes-cre*$^{Tg/+}$ and 74 male *Phf6*$^{lox/Y}$*;Nes-cre*$^{Tg/+}$. No significant difference was detected.

Checked points indicate mice that were euthanized for reasons other than hydrocephalus. Data were analysed by Mantel-Cox log-rank test.
