# Supplementary material for: Loss of PHF6 causes spontaneous seizures, enlarged brain ventricles and altered transcription in the cortex of a mouse model of the Börjeson–Forssman–Lehmann intellectual disability syndrome
Source: PLoS Genet. 2024 Oct 15;20(10):e1011428. doi: 10.1371/journal.pgen.1011428 (PMC11478892; doi:10.1371/journal.pgen.1011428)
Supplement: S8 Fig — (A) Percentage of cells expressing each cortical layer marker protein (SATB2, CTIP2, TBR1) in each of 10 pial to ventricular bins on parietal sections of three mice per genotype. No significant differences were detected between genotypes. (B) Percentage of cells for each cell type across the parietal cortex sections analysed. No significant differences were detected between genotypes. Circles represent individual mouse foetuses. N = 3 Phf6+/+ and 3 Phf6+/– foetuses. Data are presented as mean ± sem and were analysed by a two-tailed Student’s t-test. (PDF) [file pgen.1011428.s013.pdf]

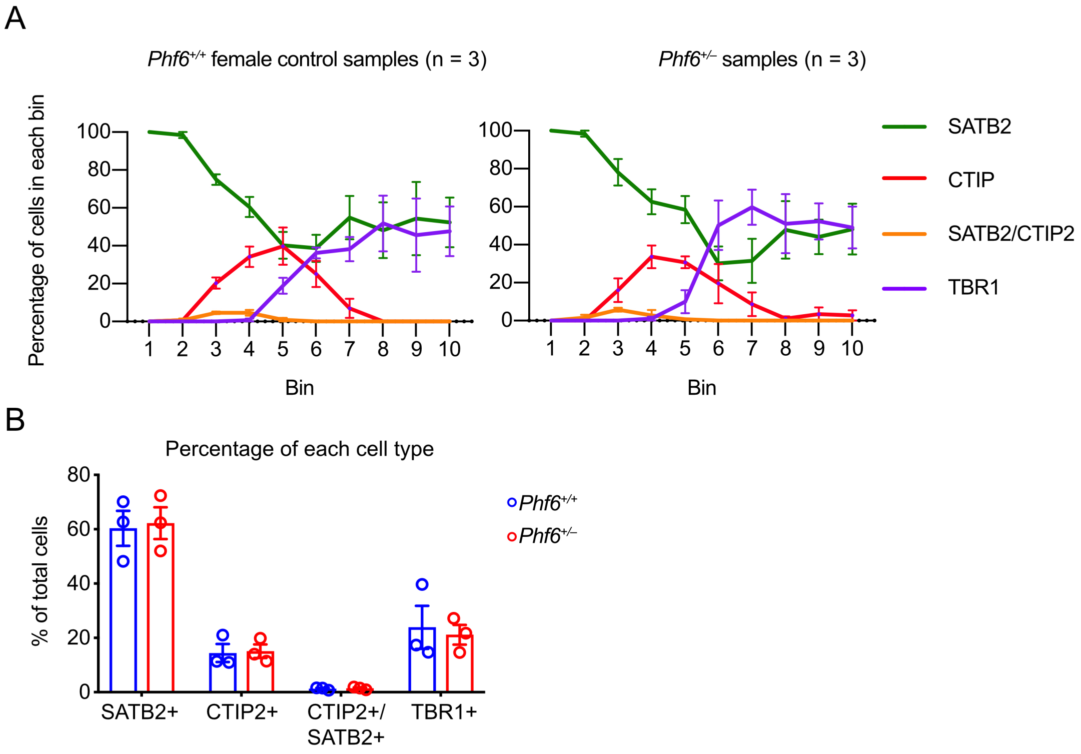

S8 Fig: Cortical layering is unaffected by heterozygous loss of *Phf6*

(A) Percentage of cells expressing each cortical layer marker protein (SATB2, CTIP2, TBR1) in each of 10 pial to ventricular bins on parietal sections of three mice per genotype. No significant differences were detected between genotypes.

(B) Percentage of cells for each cell type across the parietal cortex sections analysed. No significant differences were detected between genotypes. Circles represent individual mouse foetuses.

N = 3 *Phf6*$^{+/+}$ and 3 *Phf6*$^{+/-}$ foetuses. Data are presented as mean ± sem and were analysed by a two-tailed Student's t-test.
