# Supplementary material for: Loss of PHF6 causes spontaneous seizures, enlarged brain ventricles and altered transcription in the cortex of a mouse model of the Börjeson–Forssman–Lehmann intellectual disability syndrome
Source: PLoS Genet. 2024 Oct 15;20(10):e1011428. doi: 10.1371/journal.pgen.1011428 (PMC11478892; doi:10.1371/journal.pgen.1011428)
Supplement: S9 Fig — Representative images of immunohistochemistry staining for reelin, CUX1 (layers II-V), CTIP2 (layers V-VI) and FOXP2 (layer VI) of sections of the parietal cortex of 13-14-week-old Phf6lox/Y;Nes-creTg/+ vs. Phf6+/Y;Nes-creTg/+ mice. N = 3 mice per genotype. Scale bar = 180 μm. (PDF) [file pgen.1011428.s014.pdf]

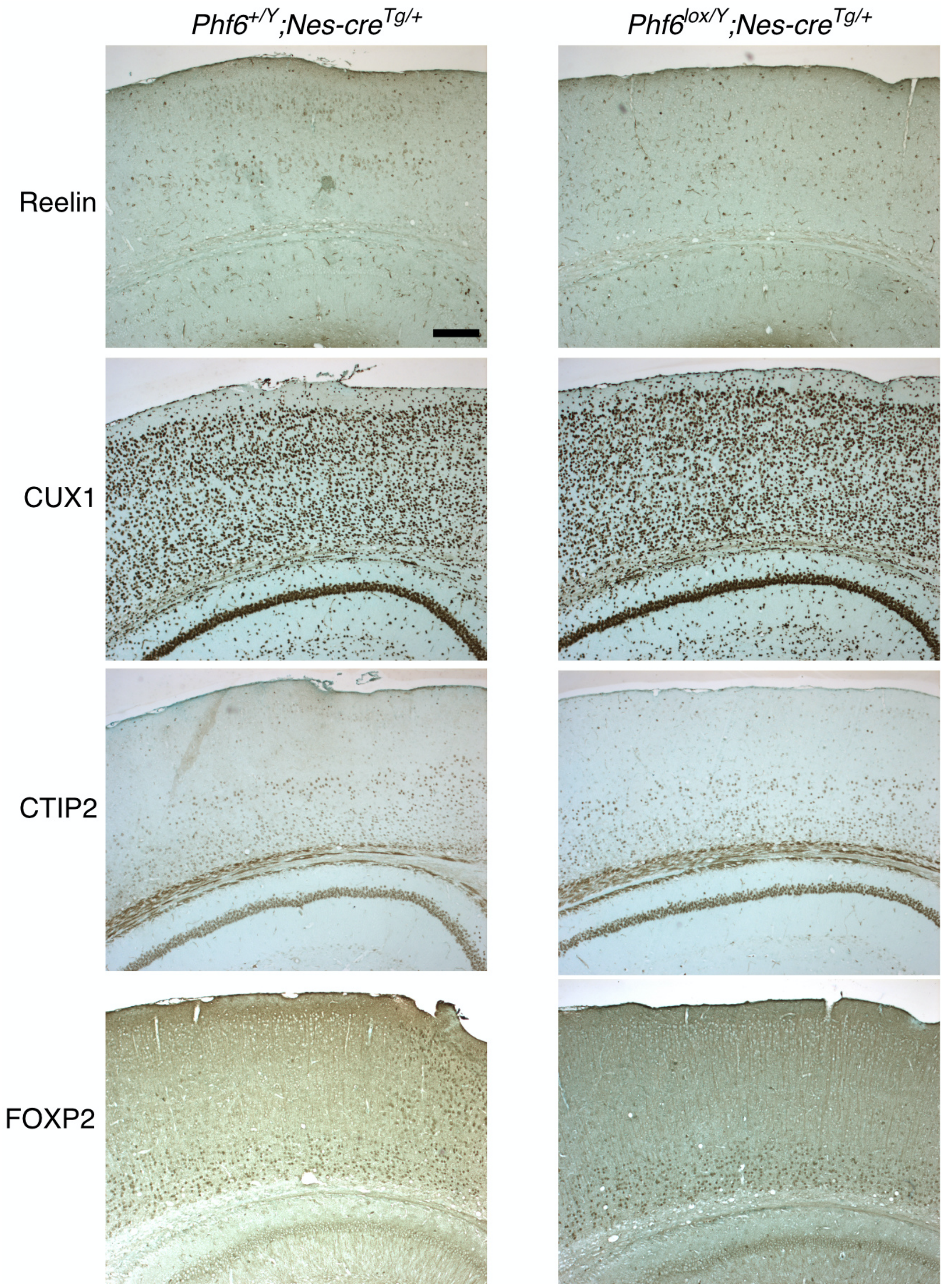

S9 Fig: Cortical layering of the adult cortex is unaffected by loss of *Phf6*

Representative images of immunohistochemistry staining for reelin, CUX1 (layers II-V), CTIP2 (layers V-VI) and FOXP2 (layer VI) of sections of the parietal cortex of 13-14-week-old *Phf6*$^{lox/Y}$;*Nes-cre*$^{Tg/+}$ vs. *Phf6*$^{+/Y}$;*Nes-cre*$^{Tg/+}$ mice. N = 3 mice per genotype. Scale bar = 180 μm.
