# Supplementary material for: Loss of PHF6 causes spontaneous seizures, enlarged brain ventricles and altered transcription in the cortex of a mouse model of the Börjeson–Forssman–Lehmann intellectual disability syndrome
Source: PLoS Genet. 2024 Oct 15;20(10):e1011428. doi: 10.1371/journal.pgen.1011428 (PMC11478892; doi:10.1371/journal.pgen.1011428)
Supplement: S10 Fig — (A) Representative images from E16.5 Phf6lox/Y;Nes-creTg/+ vs. Phf6+/Y;Nes-creTg/+ cultured cortical neurons showing staining with anti-beta III tubulin and DAPI. Scale bar = 21.25 μm. (B) Enumerations of the numbers of primary, secondary and tertiary neurites per neuron in control and Phf6-deleted cultured cortical neurons. No significant difference was detected. (C) Quantification of the length of primary, secondary and tertiary neurites in Phf6lox/Y;Nes-creTg/+ vs. Phf6+/Y;Nes-creTg/+ cultured cortical neurons. No significant difference was detected. N = 4 Phf6lox/Y;Nes-creTg/+ vs. 4 Phf6+/Y;Nes-creTg/+ foetuses. Data are displayed mean ± sem. Circles represent individual mouse foetuses. Data were analysed by using a two-tailed Student’s t-test. (PDF) [file pgen.1011428.s015.pdf]

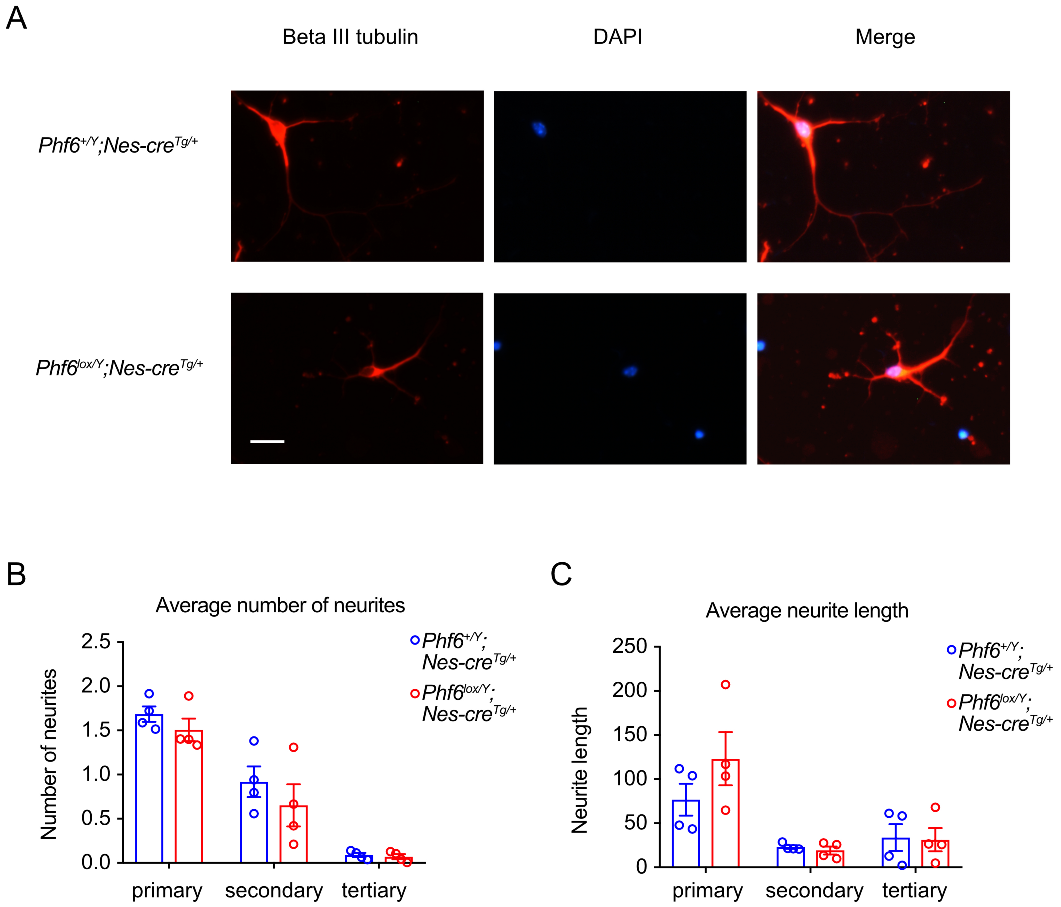

S10 Fig: Neurite length and number of cultured cortical neurons are unaffected by loss of PHF6

(A) Representative images from E16.5 *Phf6*$^{lox/Y}$*;Nes-cre*$^{Tg/+}$ vs. *Phf6*$^{+/Y}$*;Nes-cre*$^{Tg/+}$ cultured cortical neurons showing staining with anti-beta III tubulin and DAPI.

Scale bar = 21.25 µm.

(B) Enumerations of the numbers of primary, secondary and tertiary neurites per neuron in control and *Phf6*-deleted cultured cortical neurons. No significant difference was detected.

(C) Quantification of the length of primary, secondary and tertiary neurites in *Phf6*$^{lox/Y}$*;Nes-cre*$^{Tg/+}$ vs. *Phf6*$^{+/Y}$*;Nes-cre*$^{Tg/+}$ cultured cortical neurons. No significant difference was detected.

N = 4 *Phf6*$^{lox/Y}$*;Nes-cre*$^{Tg/+}$ vs. 4 *Phf6*$^{+/Y}$*;Nes-cre*$^{Tg/+}$ foetuses. Data are displayed mean ± sem. Circles represent individual mouse foetuses. Data were analysed by using a two-tailed Student's t-test.
