# Supplementary material for: Loss of PHF6 causes spontaneous seizures, enlarged brain ventricles and altered transcription in the cortex of a mouse model of the Börjeson–Forssman–Lehmann intellectual disability syndrome
Source: PLoS Genet. 2024 Oct 15;20(10):e1011428. doi: 10.1371/journal.pgen.1011428 (PMC11478892; doi:10.1371/journal.pgen.1011428)
Supplement: S11 Fig — (A,B) Cell proliferation parameters from passage 0 (primary isolation and plating) to passage 13 of neural stem and progenitor cells (NSPCs) isolated from the dorsal telencephalon of E12.5 Phf6lox/Y;Nes-creTg/+ vs. Phf6+/Y;Nes-creTg/+ embryos. (A) Cell yield at each consecutive passage after plating 50,000 cells per well of 6-well tissue culture dishes. (B) Cumulative cell growth. N = NSPC isolates from 8 embryos per genotype. Data are presented as mean ± sem (A,B). Circles represent cells isolated from individual embryos (A). Data were analysed by two-way-ANOVA with genotype and passage number as the two independent factors. (PDF) [file pgen.1011428.s016.pdf]

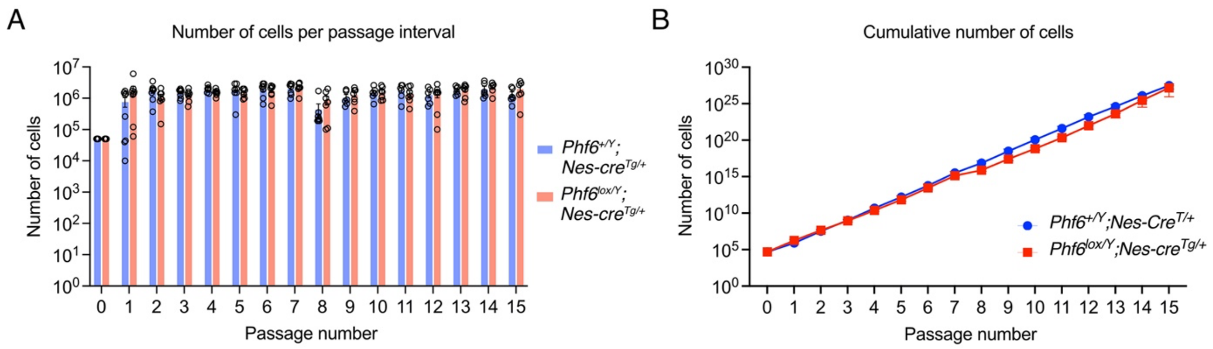

S11 Fig: Loss of PHF6 does not affect neural stem and progenitor proliferation
(A,B) Cell proliferation parameters from passage 0 (primary isolation and plating) to passage 13 of neural stem and progenitor cells (NSPCs) isolated from the dorsal telencephalon of E12.5 *Phf6$^{lox/Y}$;Nes-cre$^{Tg/+}$* vs. *Phf6$^{+/Y}$;Nes-cre$^{Tg/+}$* embryos.
(A) Cell yield at each consecutive passage after plating 50,000 cells per well of 6-well tissue culture dishes.
(B) Cumulative cell growth.
N = NSPC isolates from 8 embryos per genotype. Data are presented as mean ± sem (A,B). Circles represent cells isolated from individual embryos (A). Data were analysed by two-way-ANOVA with genotype and passage number as the two independent factors.
