# Supplementary material for: Loss of PHF6 causes spontaneous seizures, enlarged brain ventricles and altered transcription in the cortex of a mouse model of the Börjeson–Forssman–Lehmann intellectual disability syndrome
Source: PLoS Genet. 2024 Oct 15;20(10):e1011428. doi: 10.1371/journal.pgen.1011428 (PMC11478892; doi:10.1371/journal.pgen.1011428)
Supplement: S12 Fig — (A,B) Flow cytometry scatter plots of representative Phf6+/Y;Nes-creTg/+ (A) and Phf6lox/Y;Nes-creTg/+ (B) neural stem and progenitor cells (NSPCs) grown in differentiation medium (without FGF2 or EGF and with 1% FCS) for 5 days. Side scatter (SSC) and forward scatter (FSC) width (W) and height (H) were used to exclude cell doublets; SSC and FSC areas (A) were used to exclude cell debris; a fixable live/cell death marker was used to select live cells; fluorescently labelled antibodies (see methods) were used to detect astrocytes [glial fibrillary acidic protein positive cells (GFAP+) and S100 protein, beta polypeptide, neural positive (S100ß+)], oligodendrocytes (monoclonal antibody O4+) and neurons (ßIII-tubulin+). (PDF) [file pgen.1011428.s017.pdf]

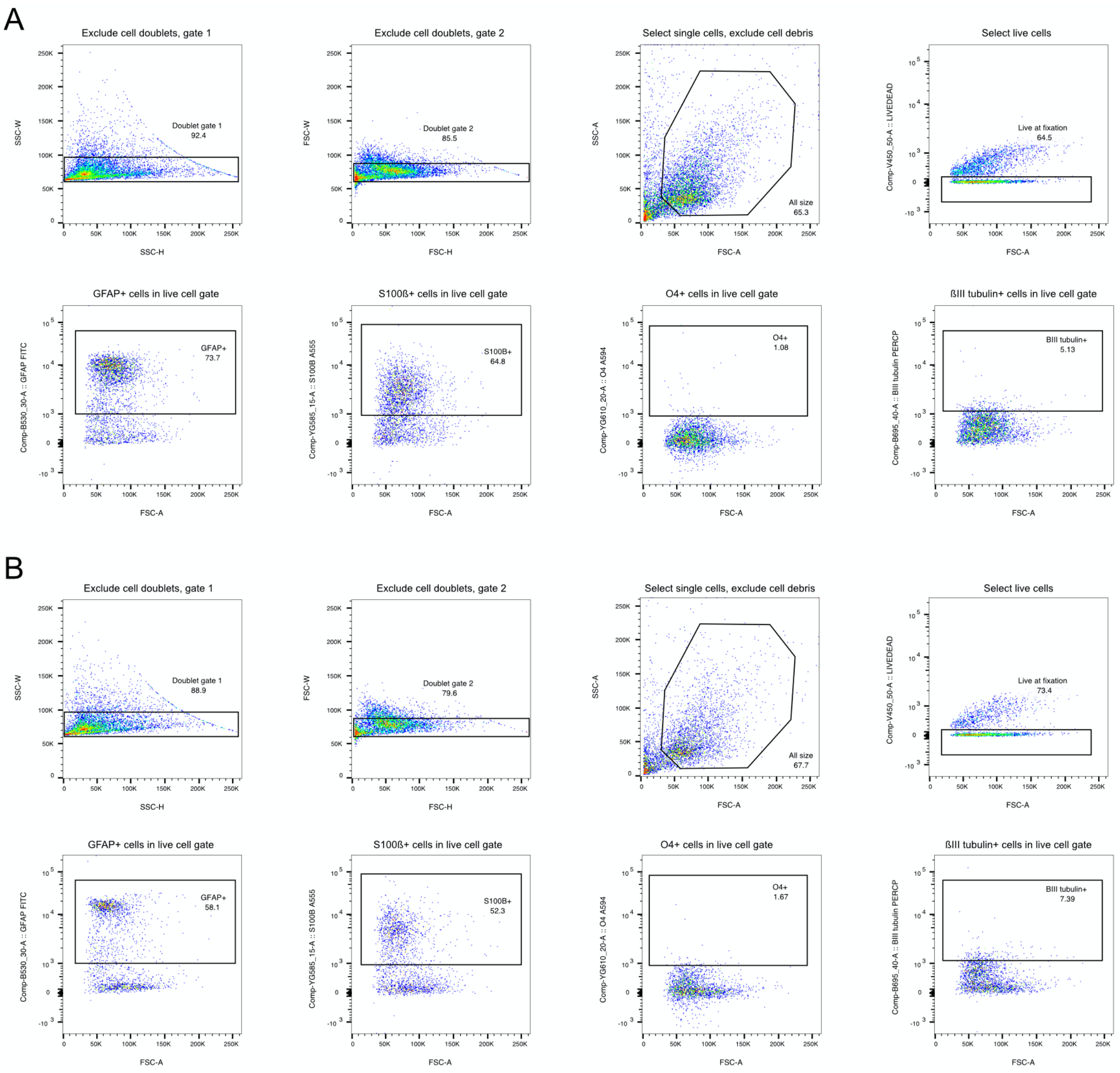

S12 Fig: Flow cytometry gating strategy to assess cell type markers in differentiating neural stem and progenitor cells

(A,B) Flow cytometry scatter plots of representative *Phf6*$^{+/Y}$*;Nes-cre*$^{Tg/+}$ (A) and *Phf6*$^{lox/Y}$*;Nes-cre*$^{Tg/+}$ (B) neural stem and progenitor cells (NSPCs) grown in differentiation medium (without FGF2 or EGF and with 1% FCS) for 5 days. Side scatter (SSC) and forward scatter (FSC) width (W) and height (H) were used to exclude cell doublets; SSC and FSC areas (A) were used to exclude cell debris; a fixable live/cell death marker was used to select live cells; fluorescently labelled antibodies (see methods) were used to detect astrocytes [glial fibrillary acidic protein positive cells (GFAP^{+}) and S100 protein, beta polypeptide, neural positive (S100ß$^{+}$)], oligodendrocytes (monoclonal antibody O4^{+}) and neurons (ßIII-tubulin^{+}).
