# Supplementary material for: Loss of PHF6 causes spontaneous seizures, enlarged brain ventricles and altered transcription in the cortex of a mouse model of the Börjeson–Forssman–Lehmann intellectual disability syndrome
Source: PLoS Genet. 2024 Oct 15;20(10):e1011428. doi: 10.1371/journal.pgen.1011428 (PMC11478892; doi:10.1371/journal.pgen.1011428)
Supplement: S13 Fig — (A) Multidimensional scaling (MDS) plot of N = 5 Phf6–/Y vs. 3 Phf6+/Y E15.5 cortices samples used for RNA-sequencing analysis, showing clustering of samples after adjustment for litter-effect; color-coded by genotype. (B) MDS plot of N = 4 Phf6lox/Y;Nes-creTg/+ vs. 3 Phf6+/Y;Nes-creTg/+ E16.5 cortical neuron isolates used for RNA-sequencing analysis showing clustering of samples after adjustment for litter-effect; color-coded by genotype. (C,D) Mean-difference plot for Phf6–/Y vs. Phf6+/Y E15.5 cortex RNA-sequencing data showing log2-fold change for each gene over average log2 expression (C). Mean-difference plot for Phf6lox/Y;Nes-creTg/+ vs. Phf6+/Y;Nes-creTg/+ E16.5 cultured cortical neuron RNA-sequencing data (D). Significantly up- and downregulated genes in the Phf6 deleted samples relative to controls are coloured red and blue respectively. NS = not significant. (E) Log2 fold-change in RNA levels of ZIC family transcription factor genes in E15.5 Phf6–/Y vs. Phf6+/Y cortex. (F) Log2 fold-change in RNA levels of the Usp18 gene in Phf6lox/Y;Nes-creTg/+ vs. Phf6+/Y;Nes-creTg/+ E16.5 cortical neurons. RNA sequencing data were analysed as described in the methods section. (PDF) [file pgen.1011428.s018.pdf]

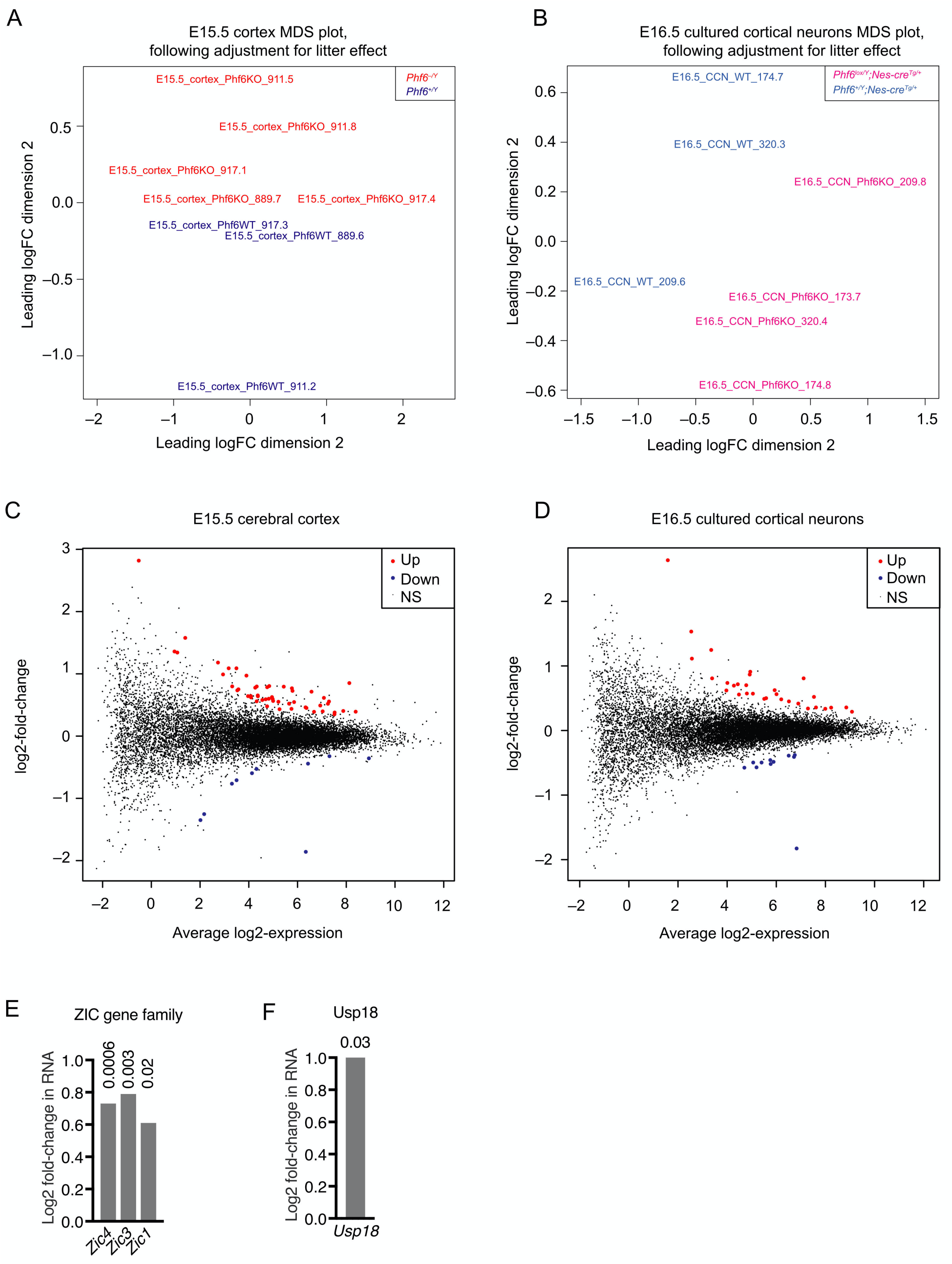

S13 Fig: RNA-sequencing analysis of *Phf6*-deleted and control E15.5 cortex and E16.5 cultured cortical neurons

(A) Multidimensional scaling (MDS) plot of N = 5 *Phf6*$^{-/Y}$ *vs.* 3 *Phf6*$^{+/Y}$ E15.5 cortices samples used for RNA-sequencing analysis, showing clustering of samples after adjustment for litter-effect; color-coded by genotype.

(B) MDS plot of N = 4 *Phf6*$^{lox/Y}$*;Nes-cre*$^{Tg/+}$ vs. 3 *Phf6*$^{+/Y}$*;Nes-cre*$^{Tg/+}$ E16.5 cortical neuron isolates used for RNA-sequencing analysis showing clustering of samples after adjustment for litter-effect; color-coded by genotype.

(C,D) Mean-difference plot for *Phf6*$^{-/Y}$ *vs. Phf6*$^{+/Y}$ E15.5 cortex RNA-sequencing data showing log_2-fold change for each gene over average log_2 expression (C). Mean-difference

plot for *Phf6*$^{lox/Y}$*;Nes-cre*$^{Tg/+}$ vs. *Phf6*$^{+/Y}$*;Nes-cre*$^{Tg/+}$ E16.5 cultured cortical neuron RNA-sequencing data (D). Significantly up- and downregulated genes in the *Phf6* deleted samples relative to controls are coloured red and blue respectively. NS = not significant.
(E) Log_2 fold-change in RNA levels of ZIC family transcription factor genes in E15.5 *Phf6*$^{-/Y}$ *vs. Phf6*$^{+/Y}$ cortex.
(F) Log_2 fold-change in RNA levels of the *Usp18* gene in *Phf6*$^{lox/Y}$*;Nes-cre*$^{Tg/+}$ vs. *Phf6*$^{+/Y}$*;Nes-cre*$^{Tg/+}$ E16.5 cortical neurons.
RNA sequencing data were analysed as described in the methods section.
