# Supplementary material for: Loss of PHF6 causes spontaneous seizures, enlarged brain ventricles and altered transcription in the cortex of a mouse model of the Börjeson–Forssman–Lehmann intellectual disability syndrome
Source: PLoS Genet. 2024 Oct 15;20(10):e1011428. doi: 10.1371/journal.pgen.1011428 (PMC11478892; doi:10.1371/journal.pgen.1011428)
Supplement: S14 Fig — (A) RT-qPCR of Phf6lox/Y;Nes-creTg/+ vs. Phf6+/Y;Nes-creTg/+ E15.5 cortex. As in the RNA-sequencing dataset, mRNA levels for the selected genes were higher in the Phf6lox/Y;Nes-creTg/+ vs. Phf6+/Y;Nes-creTg/+ cortex. (B) Representative images of RNA/RNA whole mount in situ hybridisation of E15.5 Phf6lox/Y;Nes-creTg/+ vs. Phf6+/Y;Nes-creTg/+ brains using RNA probes as indicated. N = 5–7 tissue samples per genotype. Data are presented as mean ± sem (A). Data from individual foetuses are represented as open circles (A). Data were analysed by two-way ANOVA (A). (PDF) [file pgen.1011428.s019.pdf]

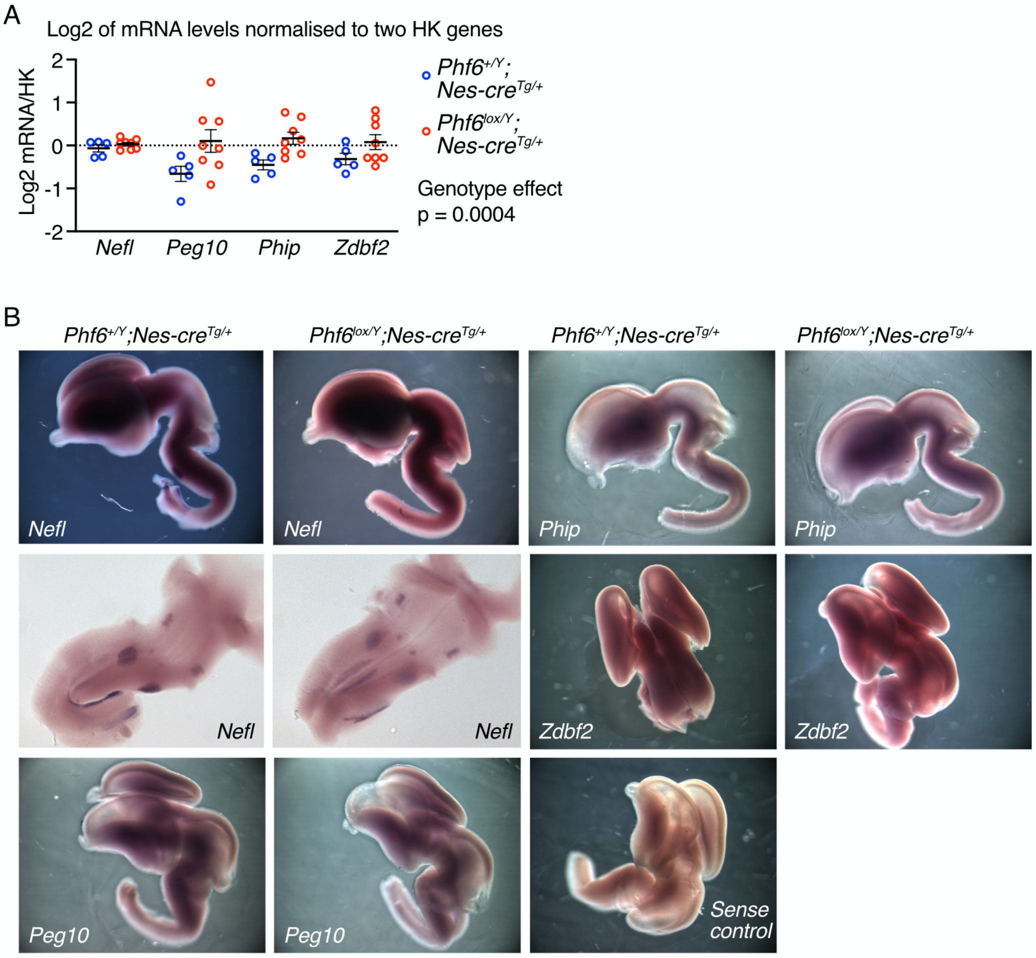

S14 Fig: Additional assessment of the expression of selected genes identified as differentially expressed between Phf6 deleted and control samples by RNA-sequencing

(A) RT-qPCR of *Phf6*$^{lox/Y}$*;Nes-cre*$^{Tg/+}$ vs. *Phf6*$^{+/Y}$*;Nes-cre*$^{Tg/+}$ E15.5 cortex.

As in the RNA-sequencing dataset, mRNA levels for the selected genes were higher in the *Phf6*$^{lox/Y}$*;Nes-cre*$^{Tg/+}$ vs. *Phf6*$^{+/Y}$*;Nes-cre*$^{Tg/+}$ cortex.

(B) Representative images of RNA/RNA whole mount in situ hybridisation of E15.5 *Phf6*$^{lox/Y}$*;Nes-cre*$^{Tg/+}$ vs. *Phf6*$^{+/Y}$*;Nes-cre*$^{Tg/+}$ brains using RNA probes as indicated.

N = 5-8 tissue samples per genotype. Data are presented as mean ± sem (A). Data from individual foetuses are represented as open circles (A). Data were analysed by two-way ANOVA (A).
