# Supplementary material for: Loss of PHF6 causes spontaneous seizures, enlarged brain ventricles and altered transcription in the cortex of a mouse model of the Börjeson–Forssman–Lehmann intellectual disability syndrome
Source: PLoS Genet. 2024 Oct 15;20(10):e1011428. doi: 10.1371/journal.pgen.1011428 (PMC11478892; doi:10.1371/journal.pgen.1011428)
Supplement: S15 Fig — (A,B) Calcium imaging results from 906 Phf6+/Y;Nes-creTg/+ and 932 Phf6lox/Y;Nes-creTg/+ cortical neurons from N = 8 Phf6+/Y;Nes-creTg/+ and 4 Phf6lox/Y;Nes-creTg/+ E16.5 foetuses. Number of peaks above background per cell per foetus in six peak height amplitude bins from 0 to <30% of total peak height (A) and 30% to maximum (B). Data are displayed as mean ± sem. Circles represent data from individual foetuses. Data were analysed by two-way ANOVA. (PDF) [file pgen.1011428.s020.pdf]

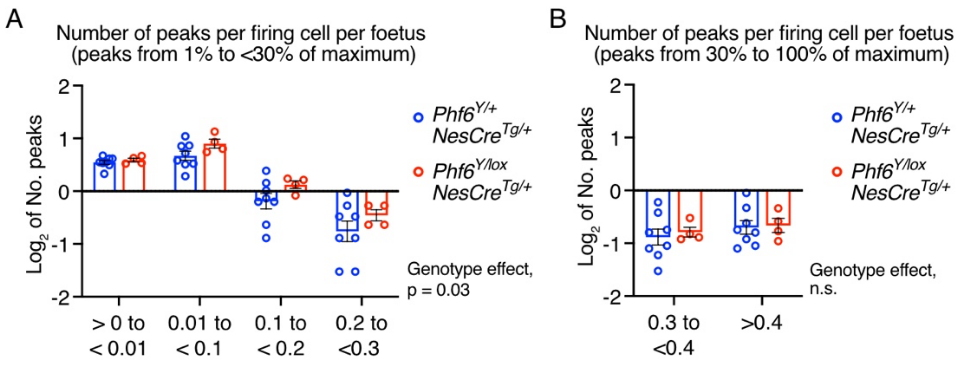

S15 Fig: *Phf6$^{+/Y}$;Nes-cre$^{Tg/+}$* cortical neurons display a higher number of calcium signalling peaks per foetus than *Phf6$^{lox/Y}$;Nes-cre$^{Tg/+}$* cortical neurons

(A,B) Calcium imaging results from 906 *Phf6$^{+/Y}$;Nes-cre$^{Tg/+}$* and 932 *Phf6$^{lox/Y}$;Nes-cre$^{Tg/+}$* cortical neurons from N = 8 *Phf6$^{+/Y}$;Nes-cre$^{Tg/+}$* and 4 *Phf6$^{lox/Y}$;Nes-cre$^{Tg/+}$* E16.5 foetuses. Number of peaks above background per cell per foetus in six peak height amplitude bins from 0 to <30% of total peak height (A) and 30% to maximum (B).

Data are displayed as mean ± sem. Circles represent data from individual foetuses. Data were analysed by two-way ANOVA.
