# Supplementary material for: Loss of PHF6 causes spontaneous seizures, enlarged brain ventricles and altered transcription in the cortex of a mouse model of the Börjeson–Forssman–Lehmann intellectual disability syndrome
Source: PLoS Genet. 2024 Oct 15;20(10):e1011428. doi: 10.1371/journal.pgen.1011428 (PMC11478892; doi:10.1371/journal.pgen.1011428)
Supplement: Attachment — Submitted filename: McRae_Leong Second responses to the reviewer.pdf [file pgen.1011428.s022.pdf]

Responses to the reviewer's comments for PLOS Genetics (PGENETICS-D-24-00726)

We would like to thank the editor and the reviewers for assessing/re-assessing out paper. Please find our point-by-point responses to this second round of reviewers' comments below

Comments to the Authors:
Reviewer #1: *In this manuscript, McRae et al. use conditional knockout mice to investigate the phenotype caused by the loss of the Phf6 gene in the mouse brain. This study helps to better understand the impact of PHF6 genetic mutations on human brain development and the pathological defects of Börjeson-Forssman-Lehmann syndrome. The authors have made improvements in the revision; however, the article still needs better organization and further analyses to make the conclusions more reliable. Some comments are listed below with the hope that the authors will find them useful.*

1. In Fig 1A, why does it show that the Ponceau S staining bands were even but beta-tubulin bands were not? This needs replicates and quantifications.

Response: We agree with the reviewer that the Ponceaus S staining indicates equal loading of the gel generated in this Western blot experiment and that the ß-tubulin bands show variable intensity.

ß-tubulin is commonly used to provide a loading control. However, when we examined the blots that we used in Figure 1A, we found that ß-tubulin was present in variable amounts in the lymphoblastoid cell lines derived from different individuals. This is the reason why we chose to also display the Ponceaus S staining of the gel.

To address the reviewer's concern, we have now added a paragraph explaining this situation better on page 26 of the second revised manuscript.

We ask the reviewer to kindly consider the fact that ß-tubulin levels in cell lines derived from Börjeson-Forssman-Lehmann syndrome individuals was not the purpose of Figure 1A. Rather, the purpose was to assess PHF6 protein levels, which are low or absent in five of the nine cell lines derived from Börjeson-Forssman-Lehmann syndrome individuals. We kindly ask the reviewer to consider the figure in this light. Ponceau S staining demonstrates equal loading. The ß-tubulin bands confirm that ß-tubulin protein is detectable in each of the samples.

The following possibilities might explain why the lymphoblastoid cell lines contain different amounts of ß-tubulin:

(a) Cells from individuals in the general population and/or Börjeson-Forssman-Lehmann syndrome individuals may result in lymphoblastoid cell lines with different levels of ß-tubulin.
(b) The production of lymphoblastoid cell lines may involve a founder effect or selection, whereby individually established cell lines may produce more or less of certain proteins, including ß-tubulin.
(c) Specific *PHF6* mutations may affect ß-tubulin levels.

We are enclosing below a graph showing the ß-tubulin content normalised to Ponceau S staining intensity in two ways:

(1) Healthy controls vs. all cell lines derived from Börjeson-Forssman-Lehmann syndrome individuals (left).
(2) Cell lines of the same genotype with respect to the *PHF6* gene mutations combined (right).

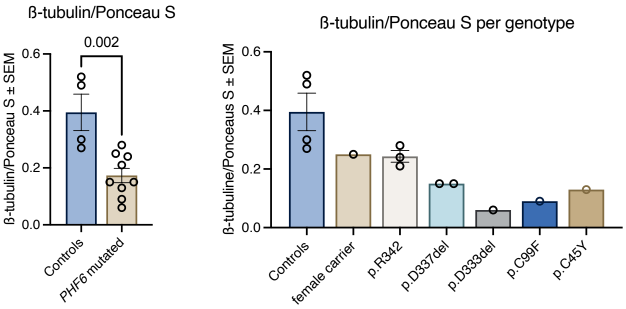

Graph (1, left) shows a significant reduction in ß-tubulin levels in *PHF6* mutant cell lines compared to healthy control cell lines. Graph (2, right) suggest that specific *PHF6* mutations cause a specific level of reduction in ß-tubulin. The segregation of the healthy control samples into two groups suggests that in addition there is either some variability in the general population or that the process of generating lymphoblastoid cell lines can result in cell lines with higher or lower ß-tubulin levels. The tight clustering of the results for the two *PHF6* mutation for which we have lymphoblastoid cell lines derived from more than one individual (p.R432*; p.D337del) suggests a specific effect of these *PHF6* mutations.

It is worth noting in this context that ß-tubulin is not the only protein to be affected by *PHF6* mutation in haematopoietic cells. We and others reported the effects of loss of PHF6 on the haematopoietic system in mice. We found, for example, *Phf6* deletion in mice resulted in a reduced number of haematopoietic stem cells and an increased number of progenitor cells, which had a greater ability to reconstitute lethally irradiated haematopoietic transplant recipients. We found that the expression of interferon response genes was upregulated in haematopoietic stem and progenitor cells in the absence of PHF6 and that combined loss of PHF6 and the interferon α and β receptor subunit 1 restored the expression levels to normal. Importantly, we demonstrated that PHF6 acts as a tumour suppressor in the lymphoid lineage. Please see McRae et al., ***Blood***. 2019; 133(16):1729-1741 for further detail. Therefore, an effect of *PHF6* mutations on the levels of specific proteins in lymphoblastoid cells is expected.

Reviewer #1: *2. In Fig 2, the quantification is about the "Time to first seizure in female mice." How about the frequency after the first seizure?*

Response: We agree with the reviewer that this is an interesting question. However, our animal ethics permits did not allow us to retain animals with tonic-clonic seizures for any length of time and so these animals were used for the brain histology presented in the paper.

Reviewer #1: *3. One of the most confusing points is that the authors claimed "Phf lox/Y;Nes-creTg/+ NSPCs formed more neurons (ßIII-tubulin+) and fewer astrocytes (GFAP+, S100ß) (Lin291)." But the cortex volume reduced (Fig 3E) with no layer neuron increase in the Phf6+/- mouse cortex (Fig S8AB). How can this be explained?*

Response: We agree that this might at first glance appear contradictory. However, as already discussed in the first revised version of the manuscript, *Phf6* deleted neural stem and

progenitor cells could undergo premature neuronal differentiation thus appearing to generate more neurons, but at the same time depleting the progenitor population and therefore over the entire period of neurogenesis generating fewer neurons because of the depletion of the stem and progenitor population. We note that we also observed a reduction in neural stem and progenitor cell self-renewal.
We would also like to ask the review to kindly consider the differences in developmental stage and cell types that display these findings that may on the surface appear contradictory. The increase in neuron formation and decrease in astrocyte formation was observed in neural stem cells isolated from E14.5 foetal forebrain. The neural stem cells represent the proliferating cell population at this point in prenatal development. The observation of the reduced cortex volume was made in animals that were adult mice; both younger and older adult mice. The developing brain undergoes substantial changes between E14.5 and adulthood and further changes during ageing. For example, all excitatory cortical neurons are fully formed by E16.5, but not all of these neurons survive. Indeed, apoptosis was a GO term enriched in *Phf6* deleted E16.5 cortical neurons. Over a period from E16.5 to well into the postnatal period, neurons that fail to make connections or experience too little neurotrophic support undergo cell death. Therefore, neuron formation during the early foetal phase is not the only process that determines the final number of neurons. Furthermore, having fewer astrocytes could also affect the volume of the cortex.

To address the reviewer's concern, we have now added a paragraph discussing these findings, which could potentially be perceived as contradictory on page 21 of the second revised manuscript.

Reviewer #1: *4. In Fig 6A,B, please add gene names in the graph. In addition, it is very surprising that only 9-10 genes were downregulated and 30-50 genes were upregulated. Only using 30-50 genes for GO analysis in Fig 6G and H is not a reliable way to get a solid conclusion. How many genes are in each pathway? Do these DEGs play a truly essential role in these pathways?*

Response: As the reviewer requested, we have now added the gene names to the heatmaps in Figure 6A and B. We note however, that the names are necessarily in very small font and that this information was and is also present in Supplemental Tables 3 and 4. If the editor considers the font too small for the gene names to be displayed in Figure 6A and B, we could add tables to Supplemental Tables 3 and 4 that specifically identify the genes displayed in Figure 6A and B.
The GO term analyses displayed in Figure 6G and H and are also listed in Supplemental Tables 3 and 4, where the detailed gene numbers are given. To further address the reviewer's question, we have now also listed the number of upregulated genes in Figure 6G and H.
We note that it is standard good practice in genomic research to identify annotation terms that are enriched in lists of differentially expressed genes, and this remains so whether the list of differentially expressed genes is long or short. For example, in Figure 6G, 22 out of the 51 up-regulated DEGs are associated with the GO term "nervous system development" and 30 are associated with "system development". These are a highly significant enrichment because the GO terms are associated with such a large proportion of the differentially expressed genes. We have not claimed that the differentially expressed genes play an *"essential role"* in any specific molecular pathway, indeed no GO analysis could ever by itself make such a strong conclusion, but the results are sufficient to correlate the differentially expressed gene results with the biological processes shown.

Reviewer #1: *5. Line 373, how are minor and major peaks defined? How does this relate to gene expression profiles and link to seizures?*

Response: Please note, that we already provided our definition of minor and major peaks in the figure legend to Figure 7.
To clarify our definition further, we have now also included the definition in the methods section on page 39 of the second revised manuscript.
We used the following cut-off for background, minor and major peaks: (1) Peaks with a height of less than 1% of the maximal peak amplitude were considered background and excluded from the analysis. (2) Peaks with a height larger than 1% and less than 10% of the maximal peak amplitude were considered minor peaks. (3) Peaks with a height larger than 10% of the maximal peak amplitude were considered major peaks.

****

Reviewer #2: *BFLS is an X-linked intellectual disability and endocrine disorder, caused by mutations in the PHF6 gene. To understand the pathogenesis of PHF6 mutations in BFLS, the authors used PBMCs from patients carrying PHF6 variants to examine PHF6 protein levels. Based on the patient results, they have generated two PHF6 mice lines: loss of Phf6 in the germline and CNS-specific deletion of Phf6. They further characterized phenotypic, anatomical, cellular and molecular changes in these mice. They found that cerebral cortex is the site of higher brain functions for cognition and decision-making. Loss of PHF6 results in the dysregulation of neuronal development and differentiation genes. Lacking of PHF6 in mice recapitulates BFLS patients in spontaneous epileptic seizures. Overall, the mice models and findings are useful in understanding the role of PHF6 in the pathogenesis of BFLS.*

Although there is a long gap of resubmission due to Covid-19, the authors had addressed most of the reviewer's concerns and the manuscript significantly improved by adding new data and revision.

Response: We thank the reviewer for the positive comment and the understanding.

Have all data underlying the figures and results presented in the manuscript been provided?
Large-scale datasets should be made available via a public repository as described in the *PLOS Genetics* data availability policy, and numerical data that underlies graphs or summary statistics should be provided in spreadsheet form as supporting information.

Reviewer #1: Yes

Reviewer #2: Yes

PLOS authors have the option to publish the peer review history of their article (what does this mean?). If published, this will include your full peer review and any attached files.

If you choose “no”, your identity will remain anonymous but your review may still be made public.

Do you want your identity to be public for this peer review? For information about this choice, including consent withdrawal, please see our Privacy Policy.

Reviewer #1: No

Reviewer #2: No

In compliance with data protection regulations, you may request that we remove your personal registration details at any time. (Remove my information/details). Please contact the publication office if you have any questions.
