# Supplementary material for: Loss of PHF6 causes spontaneous seizures, enlarged brain ventricles and altered transcription in the cortex of a mouse model of the Börjeson–Forssman–Lehmann intellectual disability syndrome
Source: PLoS Genet. 2024 Oct 15;20(10):e1011428. doi: 10.1371/journal.pgen.1011428 (PMC11478892; doi:10.1371/journal.pgen.1011428)
Supplement: Attachment — Submitted filename: McRae_Leong Responses to reviewers_02072024.docx [file pgen.1011428.s021.docx]

**Responses to the Reviewers’ Comments on PLOS Genetics (PGENETICS-D-19-01628)**

We thank the editor and the reviewers for the time and effort spent on our manuscript.

We apologise for the delay in submitting the revised version of the manuscript. We experienced considerable disruption of the work for two reasons, (a) severe COVID-19 restrictions for two years in the state of Victoria and (b) trouble breeding the *Phf6* mutant mice, which have progressive subfertility (males) and poor maternal behaviour neglecting litters. We hope that the reviewers are not troubled by this lengthy delay.

We have addressed all reviewers’ concerns with changes to the text and new experiments, which have resulted in 33 new display items (27 new figure panels; 6 expanded supplemental tables) displayed in 2 new main figures and six new supplemental figures, as well as added to existing figures. Please see our detailed responses below.

**Comments to the Authors:**

***Reviewer #1:***

*In this manuscript, the authors have engineered conditional mice to investigate the effects of Phf6 gene-loss on mouse development. This investigation is vital as a preclinical model which can lead to a better understanding of the impact of PHF6 genetic mutations in human development and, particularly, the congenital condition known as Borjeson-Forssman-Lehmann syndrome. The authors carefully conduct a series of experiments to demonstrate the hemizygous mice develop seizures, brain anatomical defects, and gene expression changes in neural (cerebral cortex) tissues. The application of cre-lox technology in mice to specifically delete Phf6 expression during early embryonic development represents one of several prominent features to this study, one which is also likely to lead to further notable publications based upon this initial finding.

Strengths of the work:
Overall, this manuscript is excellent, and will be of great interest to the readership of PLoS Genetics. There are three main strengths to this study. The first lies in the study of a nestin-cre deleted Phf6 allele to demonstrate a neural origin for seizure phenotypes in deficient mice. This finding establishes a requirement for Phf6 brain development and neuronal homeostasis.
The second strength to the study lies in the authors’ clear and careful investigation and description of the lack of overt cortical layering phenotype, as determined by immunostaining and careful quantification of data within defined regions, and across the rostro-caudal axis of mutant tissue, compared with wildtype littermates. This is an important finding to determine potential direct, causative effects of PHF6 deficiency and brain malformations, as a subset of BFLS patients have been diagnosed with cortical lamination defects, and cellular features indicative of defective neuronal maturation. Furthermore, a 2013 study using gene manipulation studies of Phf6 which employ in utero electroporation technology had highlighted a critical role for PHF6 in neuronal migration and cortical layering. More recently, cortical lamination features in the context of animal models of PHF6 deficiency were not described, despite a recent study by Cheng and colleagues (Cell Reports, 2018) investigating KO mice as well as mice with a C99F disease-associated mutation. The authors provide a balanced critique of their own work in the context of current findings.
The third strength to the work in this manuscript lies in the gene profiling studies of Phf6-deficient neural tissue and cortical neurons to elucidate gene expression networks and putative downstream target genes for future investigation. The correspondence between E15.5 cortical tissue and E16.5 mouse cortical DEX genes represents a robust and effective strategy to identify gene targets in neurons and non-neuronal cells within the cortical tissue.*

**Response:**

We thank the reviewer for the positive comments made.

***Reviewer #1:***
*Possible areas of improvement for the study:
1) It is noted that several DEX genes common to Tables 1 and 2, such as Phip and NefI, are differentially expressed in E15.5 male hemizygous KO Ccx and Nes-cre conditional male hemizygous KO E16.5 cortical neuron RNAseq datasets. Perhaps in situ hybiridisation studies could be performed to determine if changes in levels might also be accompanied by differences in the field of expression within mouse embryonic (E15.5 or E16.5) mutant tissue compared with wildtype?*

**Response:**

As the reviewer requested, we have conducted ISH experiments for differentially expressed genes in developing *NesCre;Phf6^Y/lox^* vs. *NesCre;Phf6^+/+^* brains. The new data are incorporated in Suppl Fig. 14. Because the changes in expression levels due to loss of Phf6 were subtle and therefore were unlikely to be detectable by ISH, we also performed RT-qPCR on new samples of E15.5 cortex. These additional data are displayed in Suppl. Fig. 14.

***Reviewer #1:***
*2) Could the authors comment in their Discussion section on the possibility that a birthdating (BrdU, EdU, or both) study might be informative to further demonstrate the impact, if any, of Phf6 deficiency on mouse cortical neuronal positioning?*

**Response:**

As the reviewer requested, we have added a discussion on page 19 of thymidine analogue birth dating experiments to the discussion.

***Reviewer #1:***
*3) While it is clear in Tables 1 and 2 that Phf6 transcript is lost in mutant tissue, could the authors provide an image of PHF6 immunostaining for Phf6lox/Y;NescreTg/+ brain section alongside Phf6+/lox;Nes-creTg/+ brain section to show loss of protein immunodetection?*

**Response:**

To address the reviewer’s comment, we have now included a Western blot showing the loss of PHF6 in E15.5 *r Phf6^lox/Y^;Nescre^Tg/+^* brains compared to control brains. The new data are displayed in Figure 1. In addition, we are showing RNA sequencing read coverage of *Phf6* exons, also Figure 1.

••••••••••••••••••••••••••••••••

***Reviewer #2:***

*The article by McRae et al describes several phenotypes (or lack of) in Phf6 conventional and Nestin-cre conditional knockouts. They report spontaneous seizures, enlarged lateral ventricles and different gene expression in cortex of these mice. Overall, the study does not represent any obvious advance over the recent article by Cheng and colleagues presenting a much more comprehensive and compelling phenotyping of a different strain of Phf6 deficient mice. In addition, the conditional knockout (cKO) mouse strain presented here is so poorly described that it not possible to reach any reliable conclusion from the negative results that occupy most of the manuscript. In my opinion, the article lacks essential experiments, presents others in an incomplete or inaccurate manner and contains numerous overstatements.*

**Response:**

We argue (a) that the data that *“occupy most of the manuscript”* are not *“negative results”*. Tallying up the figure panels, of 37 panels that are now displayed in the main figures, 33 (89%) show a difference between *Phf6* mutant mice and controls. Even including the supplemental data, which are typically the home of control experiments, 56 of the total of 78 display items (71%) show a difference between *Phf6* mutant mice and controls. Some of the negative data (4 display items) address findings that are controversial in the literature, including the notion that loss of PHF6 may cause cortical lamination defects, which it does not in our hands. It is surprising to find a reviewer, who, in this current science reproducibility crisis, openly propagates what is now recognised as *publication bias*, namely the refusal to publish negative data, despite the fact that it has been known for some time that such behaviour distorts scientific literature (see many papers, for example, Lancet. 337 (8746): 867–872; JAMA. 263 (10): 1385–9; eLife. 5: e21451).

We note that overall, in this study we report 71% positive and 29% negative data. However, the negative data may well be the more important data with respect to the Borjeson-Forssman-Lehmann syndrome.

Our negative results are important. They were collected in germline mutant mice, an animal model that mirrors the situation of Borjeson-Forssman-Lehmann syndrome patients better than some previous models. E.g., the first study of the role of PHF6 in cortical development using shRNA suggested a cortical neuron migration and layering defect. Does the reviewer not agree it is important to report that the germline mutants reflecting the patient mutation better do not have an obvious layering defect? We are surprised that this information is not considered important by the reviewer.

***Reviewer #2:*** *Main criticisms:
1. The article is very poorly organized. If I am not mistaken, the authors present data from (i) female mice bearing heterozygous mutations in Phf6; (ii) male mice bearing heterozygous mutations in Phf6 (i.e., Phf6 KOs); (iii) heterozygous female mice bearing a floxed Phf6 allele and a Nestin-cre transgene (i.e., heterozygous cKOs); (iv) homozygous female mice bearing two floxed Phf6 allele and a Nestin-cre transgene (i.e., nestin cKOs); and (v) male mice bearing a floxed Phf6 (i.e., also nestin cKOs). The organization of the article makes difficult to know what experiments were conducted in each of these genotypes. The presentation of the results should be streamlined; the authors should first present the results in conventional KOs and heterozygous females (which more accurately model BFLS), then the results in cKOs, and finally the comparison between the two models. This approach should allow the identification of phenotypes found only in conventional KOs (therefore originated before gene ablation in the cKOs or related to cell type no affected in the cKOs), and phenotypes observed in both models and therefore linked to the lack of Phf6 in the specific cell types affected in cKOs.*

**Response:**

We note that the first main criticism of reviewer #2 relates to the order in which we present the data. We chose to organise the paper by phenotypic topic (e.g. seizures; then histology, then cortical layering; then gene expression changes). We would find the logical flow less pleasing if we had to change to the order suggested by the reviewer, namely all results from the germline mutants first, then the tissue specific mutants. However, since it is merely changing the order we will take the editor’s advice on how to proceed.

***Reviewer #2:*** *2. Different Nestin-cre lines produce different pattern of recombination (with various degrees of mosaicism, affecting more or less cell types and triggering the recombination at different times of neural differentiation. Some reports also indicate that Nestin-cre is also expressed in non neuronal tissues such as heart, muscle, intestine, spleen, pancreas, kidney, lung and skin (Bernal and Arranz, 2018). Furthermore, the authors should keep in mind that previous studies using Nestin-cre lines have found low expression of Cre recombinase in early embryonic neural progenitors, as a result the recombination becomes efficient only in late embryonic and early postnatal periods (Liang et al., 2012). For all these reasons, it is mandatory that the authors confirm Phf6 deletion at the protein level of these cKO mice.*

**Response:**

To address the reviewer’s concern, we are now showing RNA sequencing read coverage over *Phf6* exons for germline and *Nestin*-Cre deleted samples and PHF6 protein levels in E15.5 *Phf6* mutant brains compared to control brains.

Our data show that the *Nestin*-Cre deletion is efficient and complete using Günther Schütz's *Nestin*-Cre line. No RNA sequencing reads were observed over the deleted *Phf6* exons 4 and 5. No detectable PHF6 protein can be observed in the Western blot in the *Nestin*-Cre deleted samples or in the germline deleted sample.

The new RNA read coverage graphs and new Western blot are displayed in three new figure panels in Figure 1.

We agree with the reviewer that there are a number of *Nestin*-cre-recombinase mouse lines that are unreliable. The line that we have used generated by Günther Schütz's laboratory, however, is reliable. Please see Nat Genet, 23, 99-103. This is a very well used cre-recombinase mouse strain with over 1000 citations to the original paper. In addition, we carefully tested this line using *Rosa26-loxP-stop-loxP-lacZ* reporter mice prior to starting this series of experiments.

In considering the *Nestin*-Cre results we encourage this reviewer to consider the overlap with the germline deletion. In this case, where we are trying to model BFLS patients, we are using the *Nestin*-Cre results as supporting results. They are not stand-alone results, but to be considered in conjunction with the germline mutation.

Günther Schütz's *Nestin*-Cre transgene deletes in the entire neuroepithelium of the brain and the spinal cord by E11.5 (as we had mentioned in the methods section) and all cell progeny is *Phf6* deleted, neurons and glia included. All cell types originating from this tissue are equally *Phf6* deleted. This includes interneurons, Purkinje cells, cortical pyramidal neurons.

***Reviewer #2:*** *In the line used in this study, are both glia and neurons affected or only neurons? If so, which percentages and type of neurons are affected? Moreover, there is also a great variability in the efficiency of recombination among floxed constructs. Therefore, it is absolutely essential that the authors demonstrate that their conditional knockouts really lack Phf6 in neurons.
- They reported no effect in interneurons; are interneurons affected in their nestin-cre:Phf6(f/y) mice? Do they lack Phf6?
- They reported no effect in the cerebellum; are Purkinje and granular neurons affected in their nestin-cre:Phf6(f/y) mice? Do they lack Phf6?
- They reported some (poorly characterized) effects in cortex; are cortical pyramidal neurons affected in their nestin-cre:Phf6(f/y) mice? Do they lack Phf6? And if so, in which percentage?
The whole study is basically non interpretable in the absence of this basic characterization.*

**Response:**

To address the reviewer's concerns, we are now showing RNA sequencing read coverage over *Phf6* exons showing the absence of reads over the deleted exons 4 and 5, both in *Phf6* germline deleted whole E15.5 brain and in *Nestin-*Cre deleted cortical neurons. In addition, we now show the absence of PHF6 protein in whole E15.5 brain of *Phf6* of germline deleted mice and of *Nestin-*Cre deleted mice. The new RNA sequencing read coverage graphs and new Western blot are displayed in Figure 1.

***Reviewer #2:*** *3. The most novel aspect of the study would be the characterization of neuronal-specific cKOs. Unfortunately, the authors not only fail to demonstrate the efficacy and specificity of gene ablation, in general these mice are very poorly characterized. BFLS is an intellectual disability syndrome; the authors should perform behavioral tests to demonstrate that they are modeling this condition.*

**Response:**

The efficacy of *Phf6* gene deletion both in germline deleted tissues and *Nestin-*Cre deleted tissues is now demonstrated by the absence of RNA sequencing reads over deleted *Phf6* exons and the absence of PHF6 protein in Figure 1.

Yes, indeed, we considered behavioural tests. These are lengthy experiments and will have to be the subject of a future study*.*

***Reviewer #2:*** *4. The authors consider that the observation of spontaneous seizures is one of the most important findings in this study, but they do not provide any information in methods or legends about how these numbers were obtained. Figure 2 indicates time for the first seizure. How do the authors know that this was the “first seizure”? Were the mice monitored during 24 h every day during 2 years? Are their observations based in the direct observations of the animals during cage changes (likely a few minutes per week), or was each animal observed for a given period of time every day? Did they monitor seizures incidence using surveillance cameras or implanted electrodes? Did they try to elicit seizures with sound or light?
Also, what type of seizure (tonic, clonic, absence) had the mice? And, what was the score of the seizures in the Racime scale? What was the duration of the seizures? Did the mice survive the seizure episode? Once one animal presents the first seizure, will it present recurrent seizure episodes? With what periodicity? Why is the onset of seizures so late? Because the authors could not detect earlier events or because it is a very secondary effect that can be only detected in mature and elderly mice?*

**Response:**

In response to the Reviewer’s queries, we have added the following information to the results section on page 9:

The observed seizures were grand mal seizures (tonic-clonic seizures with falling), stage 5 according to Racine.

... and the following information to the methods section on pages 15 and 26:

The mice were observed daily at specific times of the day. Mice displayed unprovoked, spontaneous seizures (no light, no sound, no other form of interference), but also seizures in response to being disturbed, for example by the cage cleaning process. In general, mice with a tendency to suffer spontaneous seizures will display seizures specifically when disturbed, e.g, during cage cleaning. Since the cage cleaning took place 2x/week, we have determined the onset of seizures within ~3.5 days accuracy. Since the latency is quite long (median latency of ~600 days), this corresponds to an accuracy of approximately ± 0.6%. The seizures lasted between 2 and 10 minutes and recurred with varying periodicity.

The seizures observed were grand mal seizures (tonic-clonic seizures with falling), stage 5 according to Racine RJ. Modification of seizure activity by electrical stimulation. II. Motor seizure. Electroencephalogr Clin Neurophysiol. 1972 Mar;32(3):281-94.

The mice survived the seizures, which took more than 2 and less than 10 minutes. The seizures were recurring with varying periodicity.

We note that we are reporting spontaneous unprovoked seizures here.

In this context, we would like to point out that this is the first report of unprovoked seizures in a mouse model of BFLS and the BFLS patients display seizures. Therefore, we think the unprovoked nature of the seizures in our model is relevant and important to report.

***Reviewer #2:***

*If the authors did not monitor continuously the mice nor properly quantified the seizures, I think that they should eliminate all the graphs presenting (unreliable) information regarding seizure onset and simply comment that spontaneous seizure episodes were observed in a percentage of the animals.*

**Response:**

We argue that our seizure data are not unreliable. As described in our answer to this reviewer’s previous question, the age of onset is determined with ± 0.6% accuracy. The reviewer might be more used to induced seizures, where a number of parameters can be collected including electrophysiology data. This is the first report of unprovoked seizures in a mouse model of BFLS and this is relevant to the patients, who also display spontaneous seizures.

***Reviewer #2:*** *5. The transcriptomic analysis seems to be poorly designed and is often over-interpreted.
- Why did the authors decide to use different time points in KOs and cKOs (E15.5 and E16.5). This decision complicates the direct comparison of the results. What was the motivation?*

**Response:**

We argue that our RNA-seq experiments are not poorly designed and, in response to the Reviewer’s query, we have added the following explanation to the methods section of the manuscript.

We used E15.5 cortex and isolated and cultured E16.5 cortical neurons for the following reasons:

The E15.5 cortex is the developing cortex at peak neurogenesis directly taken ex vivo without any inference of culture systems, but with the caveat of a mixed population of cells, including some non-neuronal cells such as blood vessel endothelial cells. Neuron cultured form E16.5 were chosen because this is the optimum stage for primary culture of cortical neurons. E16.5 neurons cultured overnight in a medium selects for neurons and provides a most homogeneous cell population of cortical neurons (>98% based on immunofluorescence marker staining).

We also used two different approaches to delete *Phf6* to determine the effects of loss of PHF6 on gene expression:

We wanted to use an inbred genetic background, C57BL/6, to keep variation due to genetic background at a minimum. We used the germline mutant to collect the E15.5 cortices. We turned to the *Nestin*-Cre deleted mice for the E16.5 neurons, when trying to collect the required number of E16.5 foetuses proved unsuccessful for 2 years for following reason:

The *Phf6* germline mutant mice on a C57BL/6 background were challenging to breed.

*Phf6^+/–^* females do not give birth naturally due to endocrine defects and so only one litter per female can be recovered by Caesarean section, giving rise to only 5 pups on average (2.5 male and 2.5 female pups on average). Only one half of the females are *Phf6^+/–^* heterozygotes (1.25 pups of the single litter on average) and so each *Phf6^+/–^* female has a replacement rate of only 1.25 *Phf6^+/–^* females per *Phf6^+/–^* females, that is if all pups survive the fostering. Therefore, the colony expands at fewer than 0.2 per mouse generation, producing 1 or 2 surplus female that can be used for foetus collection per every 10 breeding females.

***Reviewer #2:*** *- What is the overlap of the datasets obtained in KOs and cKOs? One could expect a very large overlap, but this is never presented. The authors could prepare a heatmap or scatter plot to clearly present how the differentially expressed genes (DEGs) in KOs behave in cKOs and how the DEGs in cKOs behave in KOs. Also, how the authors explain the lack of overlap between the GO analyses presented in Fig. 5E and 5F?*

**Response:**

We displayed the overlap between the effects of loss of PHF6 on E15.5 cortex and the E16.5 cultured cortical neuron in the original Supplemental Figure 9E. This panel showed a strong positive correlation between the two datasets (ROAST geneset test, p = 0.0002). To give this data point more prominence so it will not be missed, we have moved it to main Figure 6C.

The reviewer expected a large overlap between the E15.5 cortex and the E16.5 cultured cortical neuron RNA-seq datasets. As explained above, we used both sources of tissue/cells, because we expected differences and wanted to take advantage of both systems (E15.5 cortex = ex vivo, unmanipulated; E16.5 neurons cultured overnight = highly purified cell population) and wanted to control for the disadvantaged of both (E15.5 cortex = complete tissue, mostly neurons, but not exclusively; E16.5 neurons cultured overnight = subjected to cell culture, which might reveal specific cell culture dependencies on PHF6). Indeed, as we expected, these two conditions produce some different results.

We would like to point out that the fact that the two conditions show differences validates our approach, which could be seen as a strength, rather than a weakness and could recommend itself to others.

***Reviewer #2:*** *- The phenotype of KOs and cKOs is very different. If I am not mistaken (as indicated in point 1, the article should be much more explicit and clear stating these critical results), the KOs present perinatal lethality, whereas the cKOs are largely normal, can live almost two years and do not present gross anatomical alterations (except for the slight ventricle enlargement at old age, likely a consequence of the seizures) or severe phenotypes. The comparison and discussion of the results of the differential expression screens should take these essential differences in consideration. For example, are the changes exclusive of KOs responsible for the much more severe phenotype of KOs compared to cKOs?*

**Response:**

To address the reviewer's suggestion that the enlarged ventricles might be a consequence of the seizures, we have assessed the size of the ventricles in young adult mice (~9 months before the onset of seizures) and found that the ventricles are already enlarged in the *Phf6* deleted mice. The new data are displayed in a new Suppl. Fig. 6.

*Phf6* is widely expressed in the brain and outside of the brain. The fact that the *Phf6* germline mutant mice (on a C57BL/6 genetic background) die at birth, whereas the nervous-system-specific *Phf6* deleted mice (also on a C57BL/6 genetic background) live, merely suggests that the cause of death is located outside of the nervous system. It does not show that the two deletion conditions have different effects in principle on brain development. Due to the death at birth of the *Phf6* germline mutant mice, the parameters that we have assessed in the adult nervous system of *Phf6* deleted mice cannot be assessed in the germline mutant mice and so cannot be compared.

***Reviewer #2:*** *- The authors should try to validate some results of the RNA-seq screens. For example, through immunohistochemistry in brain slices of KOs and cKOs that could provide some cues about why the first ones cannot survive after birth and the second ones can develop normally.*

**Response:**

As the reviewer suggested, we have assessed some of the genes most strongly affected by loss of PHF6 by another method, RT-qPCR. We note that RNA-seq is substantially more sensitive and quantitative compared to other methods of assessment of changes in mRNA levels. The new data are in Suppl. Fig. 14.

***Reviewer #2:*** *6. The authors responded to the “Data availability” question indicating that “all data are fully available without restriction”. However, they do not indicate anywhere in the manuscript that their trancriptomics dataset has been deposited in a public database such as GEO. The authors should deposit these data and provide the access number in the manuscript. In addition, they should provide a token for access during peer review to the editor and reviewers. In most journals dealing with genomic data, the deposition of the datasets in a public repository is a mandatory requirement for publication.*

**Response:**

To address the reviewer’s concern, we are now displaying the effects of loss of PHF6 on all expressed genes (not just differentially expressed genes) in Supplemental Tables 3a,b,c,d and 4a,b,c,d.

We will be very happy to deposit any data the journal requires in the public database. We note however that releasing the RNA-seq data to a database will not provide any new information over and above what is in Supplemental Tables 3a,b,c,d and 4a,b,c,d.. It will only give the reviewer the option to perform a de novo bioinformatics analysis on our data, which is not a usual part of the reviewing process. Nevertheless, we are happy to provide FASTQ files, if the reviewer would like to receive these.

***Reviewer #2:*** *Other criticisms:
7. The Authors summary is inaccurate. The authors claim that they “report (here) the first animal model with complete loss of PHF6 from conception”. They already presented this strain in a previous publication this year (McRae et al., 2019). Furthermore, Dickinson et al. (2016) already reported that Phf6 knockouts were homozygous-lethal.*

**Response:**

We have removed the claim of priority, as requested.

***Reviewer #2:*** *8. Figure 1 presents the results of a western blot for PHF6 in cell lines derived from patients. This figure seems unnecessary (since the notion that PHF6 is missing in some patients can be directly inferred from the genetic information) and unrelated to the rest of the study. The figure could be easily eliminated. It would be, however, interesting if the authors use these cell lines to validate some of the DEGs detected in their transcriptome screens in mouse models.*

**Response:**

We consider the western blot assessing the consequences of the human *PHF6* mutations essential for our paper. It is essential to show that the human mutations result in a loss of PHF6 protein, rather than in the production of truncated protein. Truncated protein could have an abnormal function, in which case a loss of function mouse would be less suitable as a model.

Unlike the Reviewer, we do not think that inferences based on the genetic mutations are reliable. Even mutations generally considered to induce non-sense-mediated RNA decay generally retain sufficient RNA to produce truncated protein (Nat Genet. 2016 Oct;48(10):1112-8). We have now also added further results to Figure 1.

***Reviewer #2:*** *9. Also related to Figure 1: More information should be included, such as a quantification of the signal and a schematic representation of the PHF6 locus and the translated protein, indicating the position of the different mutations regarding the functional domains. Also, the signal for b-actin is saturated and therefore useless as a loading control.*

**Response:**

To address the reviewer’s concern, we are now displaying a new Western blot with a lower exposure of ß-tubulin and also Ponceau S staining as loading controls. As the reviewer requested, we have quantitated the protein levels. As the reviewer requested, we have added a schematic drawing showing where the variants are located with respect to *PHF6* exons and PHF6 protein domains. The new data are in Figure 1A.

***Reviewer #2:*** *10. Figure 3B-D: The authors investigate ventricle enlargement 17 to 37 days after seizure onset, therefore in mature or even elderly mice of a wide age range. This, together to the very low n, makes the analysis unreliable. It would be interesting that the authors investigate ventricle enlargement before the seizure onset to explore whether this change is a cause or a consequence of the seizures.*

**Response:**

As the reviewer requested, we are now showing histological sections of 13-14 weeks old brains, which show enlargement of the ventricles 9 months prior to the onset of seizures. The new data are displayed in the new Suppl. Fig. 6.

***Reviewer #2:*** *11. The most extreme example of the disorganization outlined in point 1 can be found in the section “No change in neurite number or length after culture for 5 days” (p. 9). The authors do not indicate if these cultures were obtained from conventional KOs or nestin-cKOs. If the cultures were produced from KOs (as it seems more likely), they should clearly indicate whether the embryos were females (and therefore heterozygous), males (and therefore KOs) or a mix. Again, as presented, the results cannot be interpret. Should we contrast the absence of phenotype with the severe phenotype of KO males or with the mild or absent phenotype of heterozygous and cKOs?*

**Response:**

In the main text, we referred the reader to Supplemental Figure 8 (which is now Supplemental Figure 10) and we provided all information that the reviewer was missing in that figure in our original manuscript. Which type of mutant and sex were displayed as *Phf6^lox/Y^;Nes-cre^Tg/+^* and *Phf6^+/Y^;Nes-cre^Tg/+^* in the image labelling and graph key of Supplemental Figure 8A and *8C (now Supplemental Figure 10A and 10C). The superscript “Y” indicated that the animals have a Y chromosome and so are male. The superscript “lox” indicated that the animals have a conditional *Phf6* allele. The addition of *Nes-cre^Tg/+^* indicated that the animals were positive for the *Nes-Cre* transgene. To make the sex, and genotypes clearer, we have now also added these genotypes to the figure legend (see Supplemental Figure 10).

The conditional KO is not mild within the tissue where *Phf6* is deleted, the nervous system. Like the germline mutant, the nervous-system specific mutant mice have

(1)	Seizures

(2)	enlarged lateral ventricles

(3)	gene expression changes similar to our germline mutant mice and as reported in Cheng et al.

Nothing in our data suggest that the loss of *Phf6* in the nervous system only is mild, other than that the mice survive, which places the cause of death and nothing else outside of the nervous system.

***Reviewer #2:*** *12. The authors wrote in p. 13: “indicating that upregulation of Foxp2 in Phf6-deleted E16.5 cultured cortical neurons is likely to have functional consequences”. What do they mean? They did not describe any defect in outgrowth, differentiation or neuron morphology in cKO, therefore, where is the connection? In general, the discussion of differentially expressed genes often shows a clear disconnection between the negative results presented in the manuscript and the discussed literature.*

**Response:**

To address this reviewer’s concern and to accommodate the request of reviewer #3 that we discuss the top differentially expressed genes, we have removed the discussion of *Foxp2.*

What is important to keep in mind is that most genes that are known to be required for brain development have been associated with brain development via their *loss of function* phenotype. In contrast, most genes required for brain development and affected by loss of PHF6 are *upregulated*. *Gain of function* studies are rare. In our original manuscript, we chose to discuss genes for which gain of function studies had been published. However, even where this information is available, transgenic overexpression studies typically achieve overexpression levels that exceed the increase in mRNA levels observed in the absence of PHF6. Therefore, one cannot expect to see the same level of disruption in the *Phf6* deleted mice as are observed in transgenic overexpression mice. In the case of *Foxp2,* where there are data available on the overexpression phenotype, we merely stated in our original manuscript that *Foxp2* mRNA levels were elevated in the absence of PHF6 and that an independent study found that overexpression *Foxp2* has functional consequences. The Reviewer is correct in saying that we did not find the same functional consequences that the *Foxp2* overexpression study found, likely because we did not achieve the same overexpression levels. Nevertheless, the independent study suggests that overexpression of *Foxp2* is not neutral.

***Reviewer #2:*** *Minor points:
13. Supplementary Figure 5A is missing the label of the Y-axis.
14. Supplemental Figure 6B and 6C are not cited in the text.
15. In p. 11, the citation (Corbo et al, 2002) is in a different format.
16. The format used for citations and to indicate the number of downregulated and upregulated genes is the same, which is a bit confusing (“more downregulated (104) compared to upregulated (89) genes were…”).*

**Responses to minor comments:**

13. A label has been added.

14. These Supplemental Figure panels (now 7B and 7C) have now been referred to in the text on page 11.

15. The format of the reference list has been revised.

16. This has been changed to make the number of up and downregulated genes clearer.

••••••••••••••••••••••••••••••••

***Reviewer #3:***

*This manuscript investigated the role of PHF6 (linked to BFLS) during cortical development using the knockout PHF6 mouse. They found that PH6 led to the spontaneous seizures and enlarged later ventricles. They also conducted transcriptome RNA-sequencing study to decipher the downstream molecular target genes contribute to PHF6-mediated neural development and function. The successful delivery of the study will unravel molecular mechanisms underlying the loss-of function on PHF6 in BFLS, a neural developmental disorder.

Major concern:

Majority of the results are based on the histological and morphological analysis, the lacking of the functional characterization may dampen the enthusiasm.
1. Figure 3, an experiment demonstrates whether the enlarged lateral ventricle zone is associated with abnormal neural stem cells pool, proliferation, or/and neural differentiation is important. Similarly, whether the reduced parietal cortex is associated with impaired neuronal migration, or/and neuronal formation are important to include.*

**Response:**

As the reviewer requested, we have isolated *NesCre;Phf6^Y/lox^* vs. *NesCre;Phf6^+/+^* neural stem cells (NSC), enumerated cell growth and secondary neurosphere formation and assessed their differentiation.

We already showed that cortical lamination, the result of neuronal migration during cortex development, is largely normal in *Phf6–/Y* vs. *Phf6+/Y* E18.5 foetuses (originally and now Figure 4). Additionally, we now show by immunofluorescence the position of cortical neuron subpopulations in adult *NesCre;Phf6^Y/lox^* vs. *NesCre;Phf6^+/+^* brains. The new data are displayed in the new main Figure 5, the new Supplemental Figure 11 and the new Supplemental Figure 9.

***Reviewer #3:***

*2. Apart from morphological detection, functional neuronal activity measurement, such as calcium signaling in PHF-6 KO neurons is important to investigate because BFLS is often associated with the neuronal activity changes.*

**Response:**

As the reviewer requested, we have now assessed calcium signalling a proxy for neuronal activity in cortical neurons isolated from E16.5 *Phf6^Y/lox^*; *NesCre* vs. *NesCre;Phf6^+/ +^*;*NesCre* foetuses. The new data are displayed in the new main Figure 7 and the new Supplemental Figure 15.

We hope the reviewer will be satisfied with this assessment.

***Reviewer #3:*** *Others:
1. Figure 1, needs protein level quantification. Also, beta-actin blot is overexposed.
Furthermore, amongst the mutations and truncations, which is more predominant, and which one shows an increase in adverse effects in the disease?*

**Response:**

As the reviewer requested, we have quantitated the protein levels. The new data can be found in Figure 1. To address the reviewer’s concern about the exposure of the ß-actin bands, we are providing a new western blot with ß-tubulin as a loading control, as well as a Ponceau S loading control.

We note that even the amounts of protein present are not normal PHF6 protein, as they have missense and truncating mutations. We wanted to show in the figure that a very late truncation mutation (Arg343*) does not result in almost normal PHF6 protein levels, as might have been expected, but rather nearly no detectable protein, indicating that the null mice are a reasonable model.

Interestingly, there is not a clear phenotype/genotype correlation among BFLS patients suggesting that the clinical presentation might be affected by modifier loci. This information has now been incorporated in the Discussion on page 20.

***Reviewer #3:*** *2. Figure 3, provide addition of error bars and significance in Figure 3. Also, Figure 3E needs to measure the thickness of each layer and provides quantification data.*

**Response:**

As the reviewer requested, we have added error bars to Figure 3 and measured the layer thicknesses as displayed in Figure 3E and incorporated the new data as a new figure panel in Supplemental Figure 5C.

***Reviewer #3:***

*3. Figure 4, provides explanation of reduction in brain volume but no change in cell number.*

**Response:**

We are not sure if we understand this comment correctly. Figure 4 does not address the brain volume or the cell number. The unchanged parameters displayed in Figure 4 are percentages of cell types expressing the upper layer, intermediate layer and lower layer markers, SATB2, CIP2 and TBR1. Figure 4 shows that these markers are distributed in the 10 pial to ventral bins relatively equally between genotypes and that the percentages of each of the cell types expressing these markers are unchanged. This is not to indicate that the total cell number is unchanged.

***Reviewer #3:***

*4. Are there any correlations between identified gene hits in Figure 5 with enlarged lateral ventricle volume?*

**Response:**

Based on the reviewer’s comment, we have added this information to the manuscript on page 14.

***Reviewer #3:***

*5. In depth discussion on those top 10 genes identified (Figure 5E and F) between the e15.5 cerebral cortex and e16.5 cultured cortical neuron. The convergent and divergent pathway, and how do they relate with each other involves in neural function and neural development.*

**Response:**

As the reviewer requested, we have added to the discussion of the top differentially expressed genes and their overlap between E15.5 and E16.5 on pages 22, 23 and 24. We also now display the top 10 differentially expressed genes in each of the two tissue/cell types in in a new figure panel in Figure 6D. Additionally, we moved a barcode plot showing a positive correlation between our E15.5 and E16.5 data from Supplemental Figure 13E (previously 9E) to main Figure 6 (previously 5C) to display the correlation more prominently.

It is important to note though that the phenotype information reported to associate with most genes is based on the analysis of loss of function mutants. The reporting of a gain of function phenotype is rare. The majority of differentially expressed genes due to the absence of PHF6 are upregulated. We can for example state that the loss of *Usp18* causes enlarged ventricles. However, the Phf6 mutant mice upregulate *Usp18*. Commonly, loss of function and gain of function affect the same organ systems. For example, loss of *Pax6* and overexpression of *Pax6* both affect eye development and loss and gain of *reelin* both cause cortical layering defects. However, we cannot assume that this is the case with all genes. Furthermore, the level of transgenic overexpression is often higher than the upregulation of mRNA levels that we observed in the *Phf6* deleted cells and tissue. We have included these caveats to the new discussion paragraph.

***Reviewer #3:***

*6. In the discussion section, it is mentioned that Cacna2d2 OX causes impaired axon elongation and nerve repair. In Sup Figure 8, it shows that neurite outgrowth and branching is not impaired by PHF6 loss. As supporting data, it will be good to culture cortical neurons at a longer time point and measure axon elongation of each group.*

**Response:**

We agree with the reviewer that it would be very interesting to follow up on some of the differentially expressed genes, such as *Cacna2d2.* We note that loss of PHF6 causes an increase in *Cacna2d2* mRNA levels, which may not be as pronounced as the overexpression of *Cacna2d2* in transgenic mice. The effects of loss of PHF6 on any one gene may be too small to replicate overexpression of the gene. However, loss of PHF6 affects a number of genes, and these changes in expression could combine to cause the anomalies observed in the *Phf6* null mice. To accommodate the reviewer’s request to focus the discussion on the top differentially expressed genes on pages 22, 23 and 24, we have now removed the discussion of *Cacna2d2.*

***Reviewer #3:***

*7. What is the prevalence of enlarged lateral ventricles in BFLS? Is PHF6 expression specific to brain?*

**Response:**

As mentioned in the text on page 5, dilated ventricles were identified in three of five male patients in one study (assessed by CT scan) (Ardinger, H.H., Hanson, J.W. and Zellweger, H.U. (1984) Borjeson-Forssman- Lehmann syndrome: further delineation in five cases. Am J Med Genet, 19, 653-664) and in two of three females with heterozygous PHF6 mutations as assessed by MRI (Zweier, C., Kraus, C., Brueton, L., Cole, T., Degenhardt, F., Engels, H.,

Gillessen-Kaesbach, G., Graul-Neumann, L., Horn, D., Hoyer, J. et al. (2013) A new face of Borjeson-Forssman-Lehmann syndrome? De novo mutations in PHF6 in seven females with a distinct phenotype. J Med Genet, 50, 838-847; Berland, S., Alme, K., Brendehaug, A., Houge, G. and Hovland, R. (2011) PHF6 Deletions May Cause Borjeson-Forssman-Lehmann Syndrome in Females. Mol Syndromol, 1, 294-300).

*Phf6* mRNA is present in all cells. It is more abundant during embryonic and foetal development and then declines in the postnatal period. It is difficult to detect in adult tissues by in situ hybridisation (Voss, A.K., Gamble, R., Collin, C., Shoubridge, C., Corbett, M., Gecz, J. and Thomas, T. (2007) Protein and gene expression analysis of Phf6, the gene

mutated in the Borjeson-Forssman-Lehmann Syndrome of intellectual disability and obesity. Gene Expr Patterns, 7, 858-871). PHF6 protein is detectable in adult tissues using a high-affinity antibody (McRae, H.M., Garnham, A.L., Hu, Y., Witkowski, M.T., Corbett, M.A., Dixon, M.P., May, R.E., Sheikh, B.N., Chiang, W., Kueh, A.J. et al. (2019) PHF6 regulates hematopoietic stem and progenitor cells and its loss synergizes with expression of TLX3 to cause leukemia. Blood, 133, 1729-1741).

Prompted by the reviewer’s question, we have added a description of the distribution of *Phf6* mRNA and protein to the introduction on page 6.
